# Characterization of the Mollusc RIG-I/MAVS Pathway Reveals an Archaic Antiviral Signalling Framework in Invertebrates

**DOI:** 10.1038/s41598-017-08566-x

**Published:** 2017-08-15

**Authors:** Baoyu Huang, Linlin Zhang, Yishuai Du, Fei Xu, Li Li, Guofan Zhang

**Affiliations:** 1 Key Laboratory of Experimental Marine Biology, Institute of Oceanology, Chinese Academy of Sciences, Qingdao, 266071 China; 2 Laboratory for Marine Biology and Biotechnology, Qingdao National Laboratory for Marine Science and Technology, Qingdao, 266071 China; 3 Laboratory for Marine Fisheries and Aquaculture, Qingdao National Laboratory for Marine Science and Technology, Qingdao, 266071 China; 4 National & Local Joint Engineering Laboratory of Ecological Mariculture, Institute of Oceanology, Chinese Academy of Sciences, Qingdao, 266071 China

## Abstract

Despite the mitochondrial antiviral signalling protein (MAVS)-dependent RIG-I-like receptor (RLR) signalling pathway in the cytosol plays an indispensable role in the antiviral immunity of the host, surprising little is known in invertebrates. Here we characterized the major members of RLR pathway and investigated their signal transduction a Molluscs. We show that genes involved in RLR pathway were significantly induced during virus challenge, including CgRIG-I-1, CgMAVS, CgTRAF6 (TNF receptor-associated factor 6), and CgIRFs (interferon regulatory factors. Similar to human RIG-I, oyster RIG-I-1 could bind poly(I:C) directly *in vitro* and interact with oyster MAVS via its caspase activation and recruitment domains. We also show that transmembrane domain-dependent self-association of CgMAVS may be crucial for its signalling and that CgMAVS can recruit the downstream signalling molecule, TRAF6, which can subsequently activate NF-κB signal pathway. Moreover, oyster IRFs appeared to function downstream of CgMAVS and were able to activate the interferon β promoter and interferon stimulated response elements in mammalian cells. These results establish invertebrate MAVS-dependent RLR signalling for the first time and would be helpful for deciphering the antiviral mechanisms of invertebrates and understanding the development of the vertebrate RLR network.

## Introduction

Virus infection is a major threat to all living animals and hosts have evolved broadly active mechanisms to sense viruses and suppress their proliferation. In contrast to vertebrates, invertebrates rely on innate immunity alone for virus elimination^1^; however, the antiviral mechanisms in invertebrates, such as molluscs, are far less well studied than those of vertebrates^2^. Innate immunity is the primary antimicrobial response of metazoans and relies on recognition of conserved pathogen-associated molecular patterns (PAMPs) present in microorganisms by a limited number of germline genes encoding pathogen-associated pattern recognition receptors (PRRs)^3^. Vertebrates rely mainly on the interferon (IFN) system and the induction of IFN-stimulated genes (ISGs) to counteract viruses^4^. Briefly, after virus infection, host PRRs sense viral PAMPs and initiate antiviral immune responses. These virus-activated PRRs trigger signalling cascades, leading to activation and expression of IFNs^3^. Secreted IFNs activate the JAK/STAT pathway to stimulate the expression of hundreds of ISGs^5^.

Among PRRs, the RLRs specifically detect viral RNA in the cytoplasm and induce the expression of IFNs^6^. The RLR family contains three members: RIG-I, melanoma differentiation-associated gene 5 (MDA5), and laboratory of genetics and physiology 2 (LGP2)^7–9^. RIG-I and MDA5 share a common domain organization, consisting of two tandem N-terminal caspase activation and recruitment domains (CARDs), a DExD/H-box RNA helicase domain, and a C-terminal regulatory domain (RD)^10^. CARD domains are responsible for cell signal transmission via the recruitment of downstream molecules, whereas RNA helicase and RD domains govern viral RNA binding^11^. LGP2, which lacks a CARD domain, can act as a natural negative regulator of virus-induced signalling^6^; however, it also appears to behave as a positive regulator *in vivo* in response to certain viruses through unknown mechanisms^6^. Activated RIG-I and MDA5 are predicted to interact with the mitochondrial antiviral signalling (MAVS) protein (also known as IPS-1, VISA or Cardif) through CARD-CARD interaction, and this interaction induces the recruitment of downstream signalling molecules^12–15^. MAVS is the key adaptor molecule for RLR signalling. Mammalian MAVS are usually composed of an N-terminal CARD domain, a central proline-rich region, and a C-terminal transmembrane (TM) domain. The TM domain of MAVS is essential, both for its localization to the mitochondrial outer membrane and for its induction of type I interferon^12^. Importantly, the TM domain of MAVS is also crucial in mediation of MAVS self-association and signal transduction^16, 17^. Tumour necrosis factor (TNF) receptor-associated factor (TRAF) family members are involved in RLR signalling cascades downstream of MAVS^18^ and interaction between MAVS and TRAF3 is essential for interferon regulatory factor (IRF) 3/7 activation and type I IFN production^19, 20^, whereas interaction of MAVS with TRAF2/6 is likely responsible for NF-κB activation^14^.

Among antiviral mechanisms in invertebrates, RNA interference (RNAi) is the most robust in invertebrates, especially insects and nematodes^21^. Aside from RNAi-mediated sequence-specific antiviral responses, a protein-based antiviral immune mechanism may also exist in some invertebrates^2^. One effector of antiviral responses in invertebrates is Vago, which appears to function as a cytokine, similar to mammalian IFN^22–24^. However, whether invertebrate RLRs or TLR homologs can recognize non-specific nucleic acids to activate antiviral cytokines and the details of the associated antiviral signalling transduction pathways are largely unknown and require further investigation.

The Pacific oyster (*Crassostrea gigas*) is a representative bivalve mollusc and lophotrochozoan protostome in which the antiviral immune mechanisms are poorly understood^25–27^. As a sessile filter feeder living in estuaries and intertidal zones, the oyster is constantly exposed to a wide range of pathogens, making it an attractive model for study of the invertebrate innate immune system. Oysters are widely distributed and support major aquaculture and fishery industries worldwide^28^; however, recent mass mortality of oysters caused by ostreid herpesvirus 1 (OsHV-1) has severely affected Pacific oyster production^29–35^. Hence, there is an urgent need to better understand the immune mechanisms of oysters to facilitate development of new strategies to control OsHV-1.

Efforts have been made to elucidate the mechanisms of innate immunity in oysters and some progress has been made. Autophagy has been shown to play an important role in oyster antiviral immunity^36^. Moreover, poly(I:C) can induce a protective antiviral immune response against subsequent challenge with OsHV-1 in the Pacific oyster, suggesting that these organisms may be able to recognize non-specific nucleic acids^37^. Further research is needed to explore the PRRs that recognize virus PAMPs and the associated antiviral signalling pathways to elucidate the antiviral mechanisms of molluscs. Of note, the oyster genome is predicted to encode several evolutionarily conserved nucleic acid sensors and their downstream signalling molecules, including RLRs, TLRs, TRAF family members, and IRFs^38, 39^. Moreover, the transcription of many of these genes is induced after virus challenge^26, 40–42^; however, further detailed investigation is required before it is possible to conclude that oysters possess sophisticated and complex RLR or TLR antiviral signalling pathways. Furthermore, the functions of genes involved in invertebrate antiviral signalling pathways remain poorly studied. In this study, we characterized for the first time a functional MAVS-dependent RLR antiviral signalling pathway in the oyster. We identified a number of key genes in the Pacific oyster*, Crassostrea gigas*, homologous to those involved in the mammalian RLR antiviral signalling pathway, including the first cloned invertebrate *MAVS* gene. We focused on the roles of these genes in host responses to OsHV-1 and poly(I:C) challenge and elucidated details of the oyster RIG-I/MAVS signal transduction pathway. Functional studies of these novel molecules will be helpful for revealing the antiviral mechanisms in invertebrates and understanding the original formation of the RLR pathway of vertebrates.

## Results

### The antiviral response of RLR pathway genes after virus infection of oyster larvae

**Figure 1:**
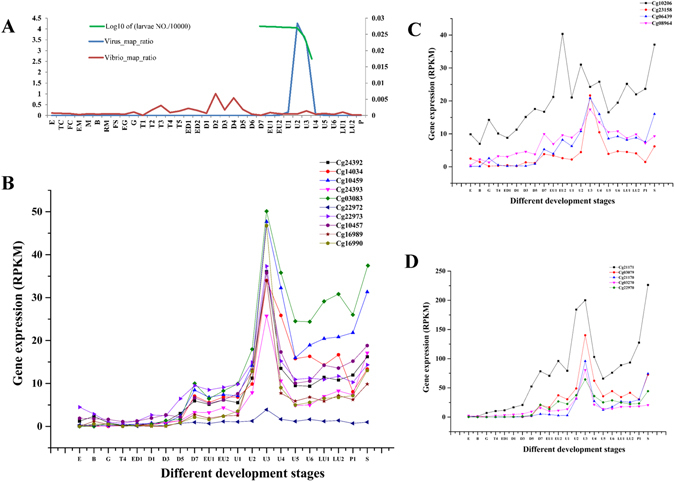
Response of oyster larvae to OsHV-1 infection. (**A**) Ratio of RNA-seq reads mapping to the OsHV-1 and *Vibrio* genomes; the number of oyster larvae is also shown. Sampling times of oyster larvae and abbreviations for developmental stages are the same as those in Table S12 of ref. 31. The abbreviations are: E: Egg; TC: Two cells; FC: Four cells; EM: Early morula; M: Morula; B: Blastula; RM: Rotary movement; FS: Free swimming; EG: Early gastrula; G: Gastrula; T: Trochophore; ED: Early D-shape larvae; D: D-shape larvae; EU: Early umbo larva; U: Umbo larva; LU: Later umbo larva; P: Pediveliger. S: Spat. (**B**) Expression of RIG-I like receptors (RLRs) in oyster larvae infected with OsHV-1. *Cg24392*, *Cg14034*, *Cg22973*, and *Cg03083* are RLRs with CARD domains, and the remaining genes do not possess CARD domains. (**C** and **D**) Expression of genes involved in the RLR signalling pathway, including *TRAF2* (*Cg08964*), *TRAF3* (*Cg23158*), *TRAF6* (*Cg10206*), three *MITA* (transmembrane protein 173) genes (*Cg06439*, *Cg03079*, and *Cg22970*), and three *IRF* genes (*Cg21170*, *Cg21171*, and *Cg03270*).

Characterization of transcriptomic responses to virus infection is an effective approach to reveal mechanisms of antiviral immunity. By analysing the transcriptome of oyster larvae infected with OsHV-1 at different developmental stages (Fig. 1A), we found that numerous genes in the RLR pathway were differentially regulated in response to viral challenge. RLRs are a family of cytoplasmic antiviral sensors^43^ and there is evidence that the oyster genome may encode more than ten of these proteins^39^. We chose ten oyster RLRs that we had confirmed by PCR amplification, and analysed their expression profiles in virus-infected oyster larvae (Fig. 1B). The results demonstrated that when larvae were infected at the U1 stage, the expression of all oyster RLRs was upregulated, and although the changes in gene expression levels were not completely uniform, they all peaked at the same time in the U3 stage.

The expression patterns of other genes that participate in the RLR pathway were also investigated (Fig. 1C,D). Briefly, expression of almost all of the test genes was induced by OsHV-1 virus challenge, with expression peaking at the U3 stage, except for *CgTRAF6*, whose expression peaked earlier, at the EU2 stage. Additionally, the expression of three *CgIRF* genes (*Cg21170*, *Cg21171*, and *Cg03270*) and two *CgMITA* genes (*Cg03079* and *Cg22970*) was highly upregulated in response to viral challenge. In contrast, the expression of these genes in oyster larvae that developed normally and without a virus challenge did not exhibit the same upregulation or similar expression profiles (Supplementary Figs 1 and 2).

### Identification of key genes in the oyster RLR antiviral signalling pathway

Almost all of the key genes of the RLR signalling pathway have been predicted in the genome of the Pacific oyster^38^. There is also evidence of massive expansion and functional divergence among oyster innate immunity genes^39^. To better understand the function of the oyster RLR signalling pathway, we first cloned a number of key oyster genes homologous to those encoding mammalian RLR antiviral signalling pathway proteins, including *CgRIG-I-1* (*Cg24392*, GenBank Accession number: KY630188), *CgMAVS* (GenBank Accession number: KY630189), *CgTRAF2* ^44^ (*Cg08964*), *CgTRAF3* ^45^ (*Cg23158*), *CgTRAF6* (*Cg10206*, GenBank Accession number: KY630190), and two oyster *IRF* genes, *CgIRF2* (*Cg21171*, GenBank Accession number: KY630191) and *CgIRF8* (*Cg03270*, GenBank Accession number: KY630192), (see Supplementary data for sequence details) from the genome of *C. gigas*. Importantly, this includes the first reported cloning of a *MAVS* gene from an invertebrate. As MAVS is the most important adaptor in the RLR antiviral signalling pathway, the identification of a *MAVS* gene in oyster suggests that invertebrates may possess a functional RLR antiviral pathway.

### Sequence and structural analysis of CgRIG-I-1 and CgMAVS

**Figure 2:**
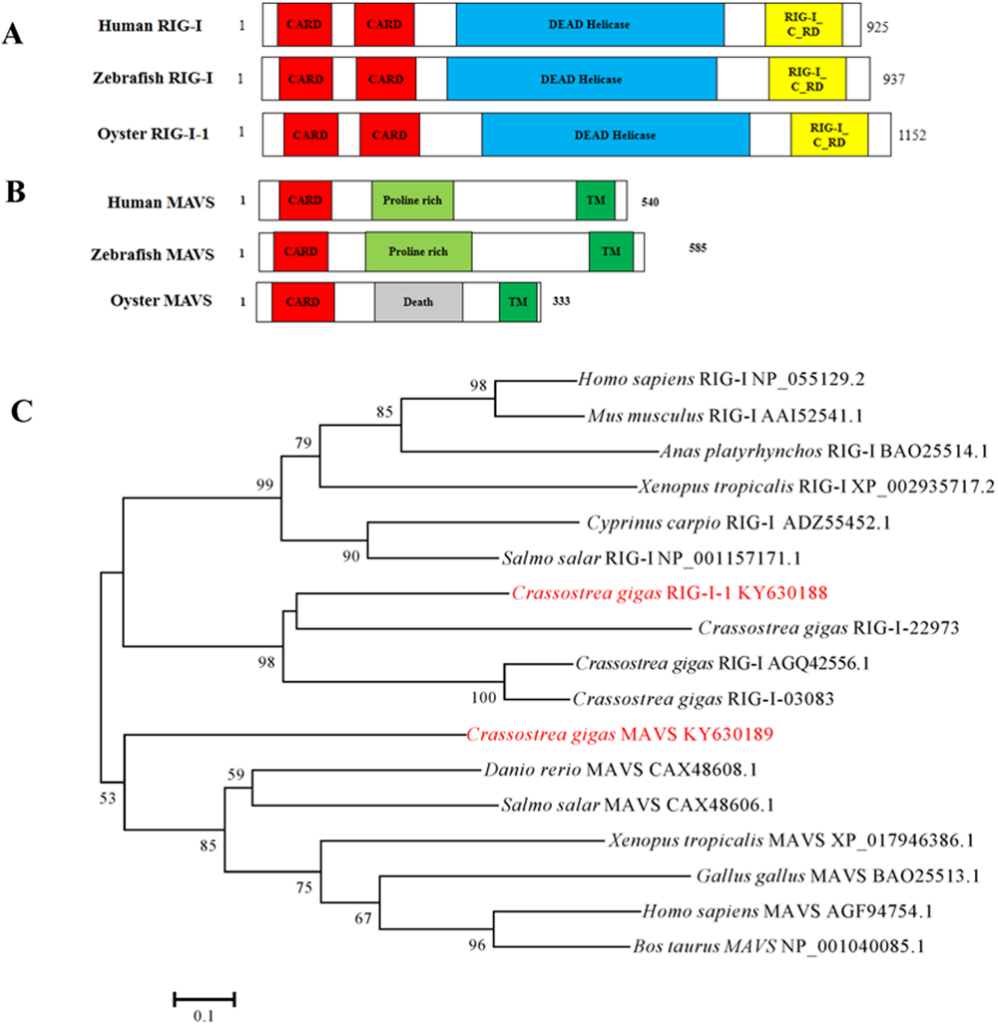
Sequence analysis of CgRIG-I-1 and CgMAVS. (**A**) Domain topology of CgRIG-I-1 compared with human RIG-I and zebrafish RIG-I. CARD: Caspase activation and recruitment domain, RIG-I-C-RD: C-terminal regulatory domain of RIG-I. (**B**) Domain topology of CgMAVS compared with human MAVS and zebrafish MAVS. TM: Transmembrane domain. (**C**) Neighbour-joining tree of RIG-I and MAVS was constructed using protein sequences of CARD domains. The numbers at nodes indicate bootstrap values. The phylogenetic tree was built with Mega v5.05. The neighbour-joining method was used to calculate the trees, with 1000 bootstrap tests.

A full-length cDNA of 4502 bp was isolated from an oyster cDNA library and designated *CgRIG-I-1*. *CgRIG-I-1* encodes a polypeptide of 1152 amino acids and SMART analysis of its predicted domain structure indicated the presence of two CARD domains, an RNA helicase domain and a C-terminal regulatory domain, consistent with the structures of human and zebrafish RIG-I (Fig. 2A). We also identified the first invertebrate *MAVS-*like gene in the Pacific oyster, *CgMAVS*. Similar to vertebrate MAVS, the CgMAVS protein is predicted to possess N-terminal CARD and C-terminal transmembrane (TM) domains (Fig. 2B). Sequence analysis demonstrated that the CARD domain of CgMAVS shares approximately 26% and 28% identity with those of the human and zebrafish MAVS proteins, respectively. However, CgMAVS also possesses a Death domain after its CARD domain, which is different from the proline-rich domains found in human and zebrafish MAVS proteins, suggesting that CgMAVS may recruit molecular partners different from those involved in mammalian MAVS signal transduction.

A phylogenetic tree was constructed based on the CARD domain of RIG-I and MAVS from human, mouse, chicken, *Xenopus*, fishes, and oysters (Fig. 2C). The phylogenetic tree suggests that the CARD domain of MAVS is different from those of RIG-I. The phylogenetic tree also shows that CgMAVS is the ortholog of vertebrate MAVS. As the first identified invertebrate *MAVS* gene, *CgMAVS* may be an ancestral molecule of mammalian *MAVS*, and vertebrate *MAVS* genes may have originated from invertebrate *MAVS* through gene duplication events and functional refinement. Additionally, four oyster RIG-Is with CARD domains form a cluster, which is divergent from that of vertebrate RIG-Is.

### CgRIG-I-1 responds to virus PAMP challenge and binds poly(I:C) directly

**Figure 3:**
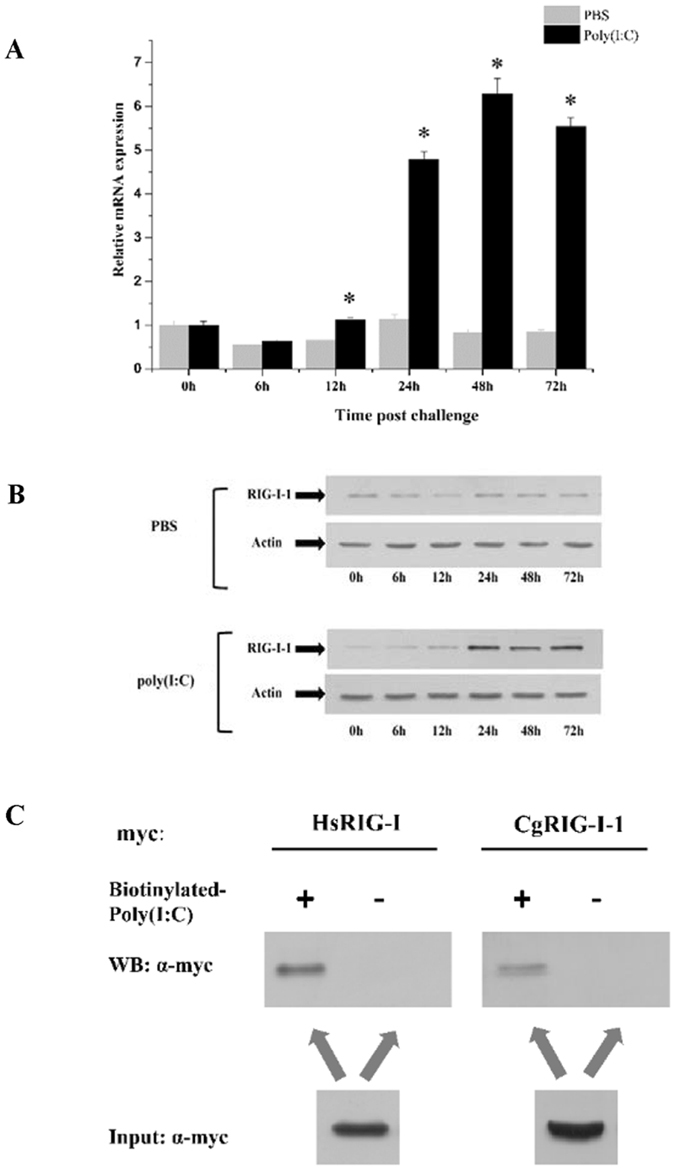
CgRIG-I-1 responds to poly(I:C) challenge and binds poly(I:C) *in vitro*. (**A** and **B**) Expression profiles of CgRIG-I-1 after poly(I:C) challenge analysed by quantitative real-time PCR (qRT-PCR) and western blot, respectively. qRT-PCR was performed in triplicate for each sample using the β-actin gene as an internal control. Expression levels were determined using the 2^−ΔΔCT^ method. The expression level at 0 h post PBS injection was set as baseline (1.0). Vertical bars represent the mean ± SD (N = 3). *P < 0.05, between challenged and control at the same time point. (**C**) Poly(I:C) pull-down assay demonstrating that CgRIG-I-1 can bind poly(I:C) *in vitro*.

Previous studies have demonstrated that *CgRIG-I-1* mRNA expression is upregulated after OsHV-1 challenge in oyster spat^26^. Our analysis found that *CgRIG-I-1* also participates in antiviral immunity in oysters at the larval stage (Fig. 1B). In this study, poly(I:C), a synthetic double-stranded RNA (dsRNA) viral analogue that can induce protective antiviral immune responses in oyster against subsequent viral challenge, was used to stimulate the oysters. Quantitative RT-PCR and western blotting demonstrated upregulation of CgRIG-I-1 in response to poly(I:C) challenge (Fig. 3A and B).

As a PRR, human RIG-I can directly interact with poly(I:C) in the cytoplasm^7^. CgRIG-I-1 also locates in the cytoplasm (Supplementary Fig. 3). Therefore we speculated that CgRIG-I-1 may also bind poly(I:C) directly. To test this hypothesis, CgRIG-I-1-myc was expressed in HEK293T cells and subjected to poly(I:C) pull-down assays, with human RIG-I as a positive control. As a result, we detected an interaction between CgRIG-I-1 and poly(I:C), indicating that CgRIG-I-1 may act as a PRR and that the recognition of PAMPs may be crucial for triggering a downstream signalling cascade (Fig. 3C).

### CgMAVS is key adaptor protein of oyster RLR pathway

**Figure 4:**
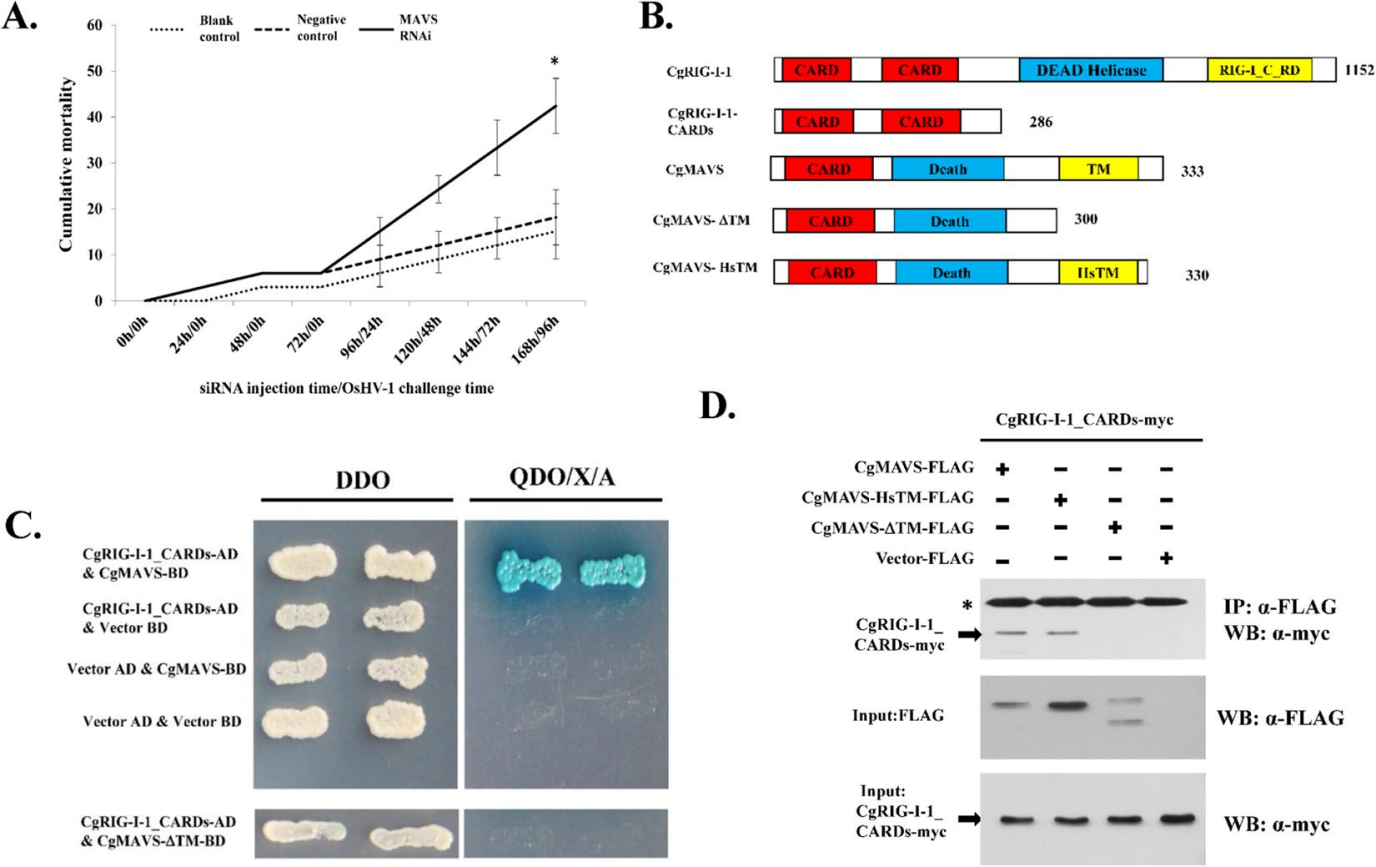
CgMAVS is involved in the antiviral immunity of the oyster and interacts with the CARD domains of CgRIG-I-1. (**A**) RNA interference of CgMAVS led to increased sensitivity of oysters to OsHV-1 infection. Healthy oysters were injected with PBS (control), CgMAVS-, or GFP-siRNA (negative control) and 72 h later challenged with 10^6^ OsHV-1 virus particles and cumulative mortality recorded every 24 h after virus challenge. (**B**) Names and schematic representations of the full-length and truncated proteins used in this study. HsTM: transmembrane domain of human MAVS. (**C** and **D**) Interaction between CgMAVS and CgRIG-I-1 was detected by yeast two hybrid (Y2H) (**C**) and co-immunoprecipitation (Co-IP) (**D**) assays. DDO: -Leu/-Trp double-dropout media, QDO/X/A: -Ade/-His/-Leu/-Trp quadruple dropout media with X-α-Gal and aureobasidin. The transmembrane domain of CgMAVS is important in mediating this interaction.

MAVS is the crucial adaptor protein in the RLR antiviral signalling pathway. It can interact with RIG-I via its N-terminal CARD domain to transduce immune signals and activate NF-κB and IRF3^12^. To better understand the role of CgMAVS in antiviral immunity, we first performed *CgMAVS* RNA interference (RNAi) experiments. After knockdown of *CgMAVS* expression (Supplementary Fig. 4), OsHV-1 was injected into the muscles of oysters, and their mortality rates were recorded (Fig. 4A). The results demonstrated that suppression of *CgMAVS* significantly increased oyster mortality after OsHV-1 infection, suggesting that MAVS plays an important role in oyster antiviral immunity.

As CgMAVS also possesses an N-terminal CARD domain and localizes in cytoplasm (Supplementary Fig. 3), we used yeast two-hybrid (Y2H) and co-immunoprecipitation (Co-IP) assays to investigate potential interactions between CgMAVS and CgRIG-I-1. Initial experiments did not detect any interaction between the full-length CgRIG-I-1 and CgMAVS proteins by Y2H or Co-IP (data not shown). Therefore, we investigated the relationships between the CARD domains of CgRIG-I-1 and various deletion mutants of CgMAVS (Fig. 4B). In Y2H assays, growth and coloration tests indicated that the CARD domains of CgRIG-I-1 were capable of interacting with full-length CgMAVS; however, a CgMAVS protein lacking the transmembrane domain failed to bind the CARD domains of CgRIG-I-1 (Fig. 4C). Co-IP assays confirmed the Y2H results. Furthermore, when we replaced the TM domain of CgMAVS with that of human MAVS, the interaction between CgRIG-I-1 CARDs and CgMAVS was rescued (Fig. 4D).

**Figure 5:**
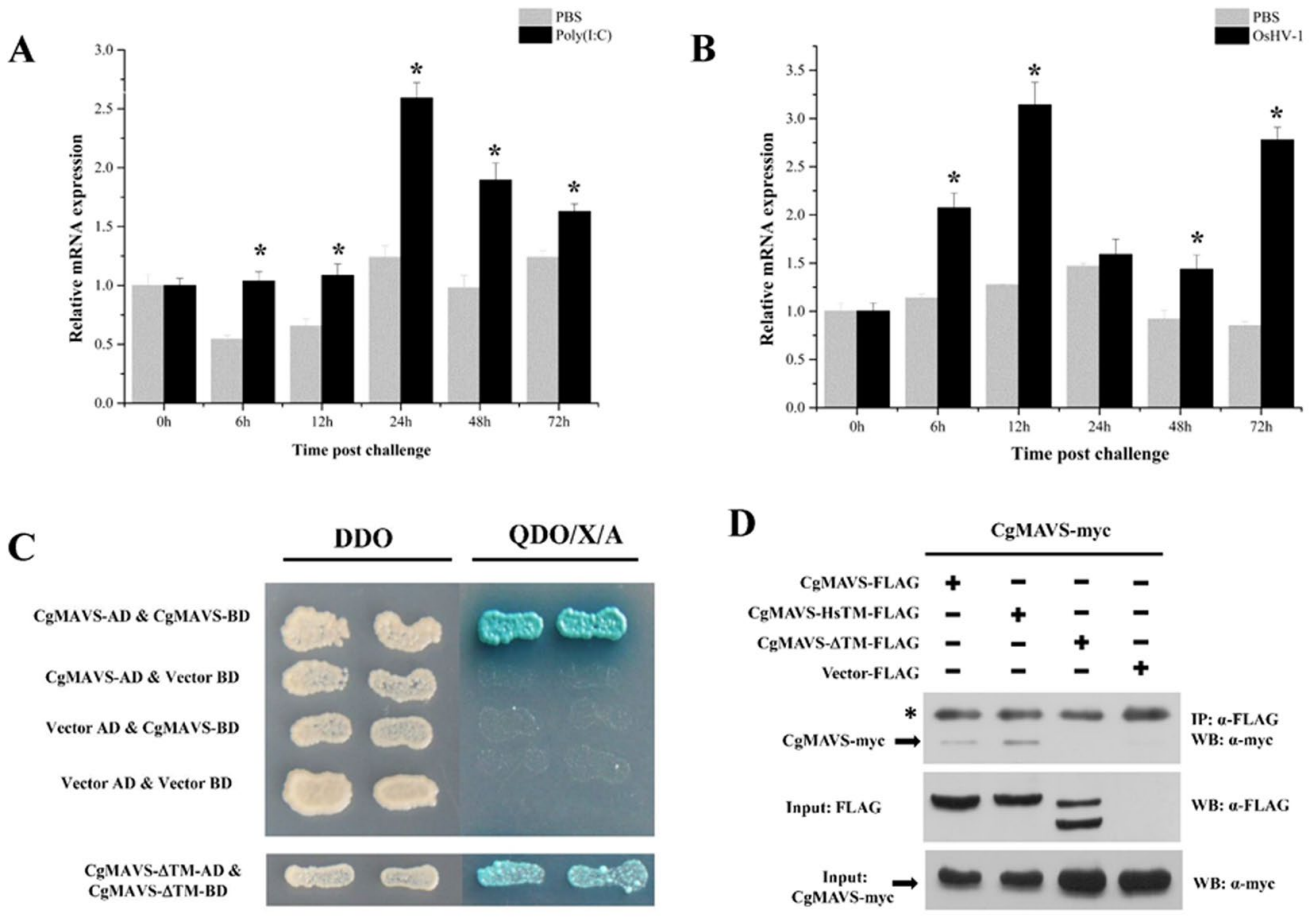
CgMAVS responds to poly(I:C) and OsHV-1 challenges and forms homodimers. **(A** and **B**) Expression profile of CgMAVS after poly(I:C) and OsHV-1 challenges analysed by qRT-PCR. (**D** and **E**) Dimerization of CgMAVS was analysed by Y2H (**D**) and Co-IP (**E**). DDO: -Leu/-Trp double-dropout media, QDO/X/A: -Ade/-His/-Leu/-Trp quadruple-dropout media with X-α-Gal and aureobasidin. The TM domain of CgMAVS was important in mediating this interaction.

To further investigate the immunological significance of CgMAVS, we performed qRT-PCR to determine the transcription levels of *CgMAVS* in response to poly(I:C) and OsHV-1 challenges (Fig. 5A and B). Although *CgMAVS* was not dramatically upregulated, its response to the virus and poly(I:C) challenges suggests that CgMAVS may function in oyster antiviral immunity.

The TM-dependent self-association of human MAVS is crucial in antiviral innate immune signalling^16^. We speculated that CgMAVS may also form homodimers. Y2H and Co-IP assays were employed to detect self-association of CgMAVS. In the Y2H assay, growth and coloration tests indicated that CgMAVS could form dimers. Additionally, experiments using CgMAVS lacking the TM domain indicated a clear attenuation of the self-association (Fig. 5C). The co-IP experiments confirmed the Y2H results. Furthermore, when we replaced the TM domain of CgMAVS with that of human MAVS, an interaction between Myc-tagged CgMAVS and FLAG-tagged CgMAVS-HsTM was detected (Fig. 5D).

### CgTRAF6 binds CgMAVS and specifically activates the NF-κB pathway

**Figure 6:**
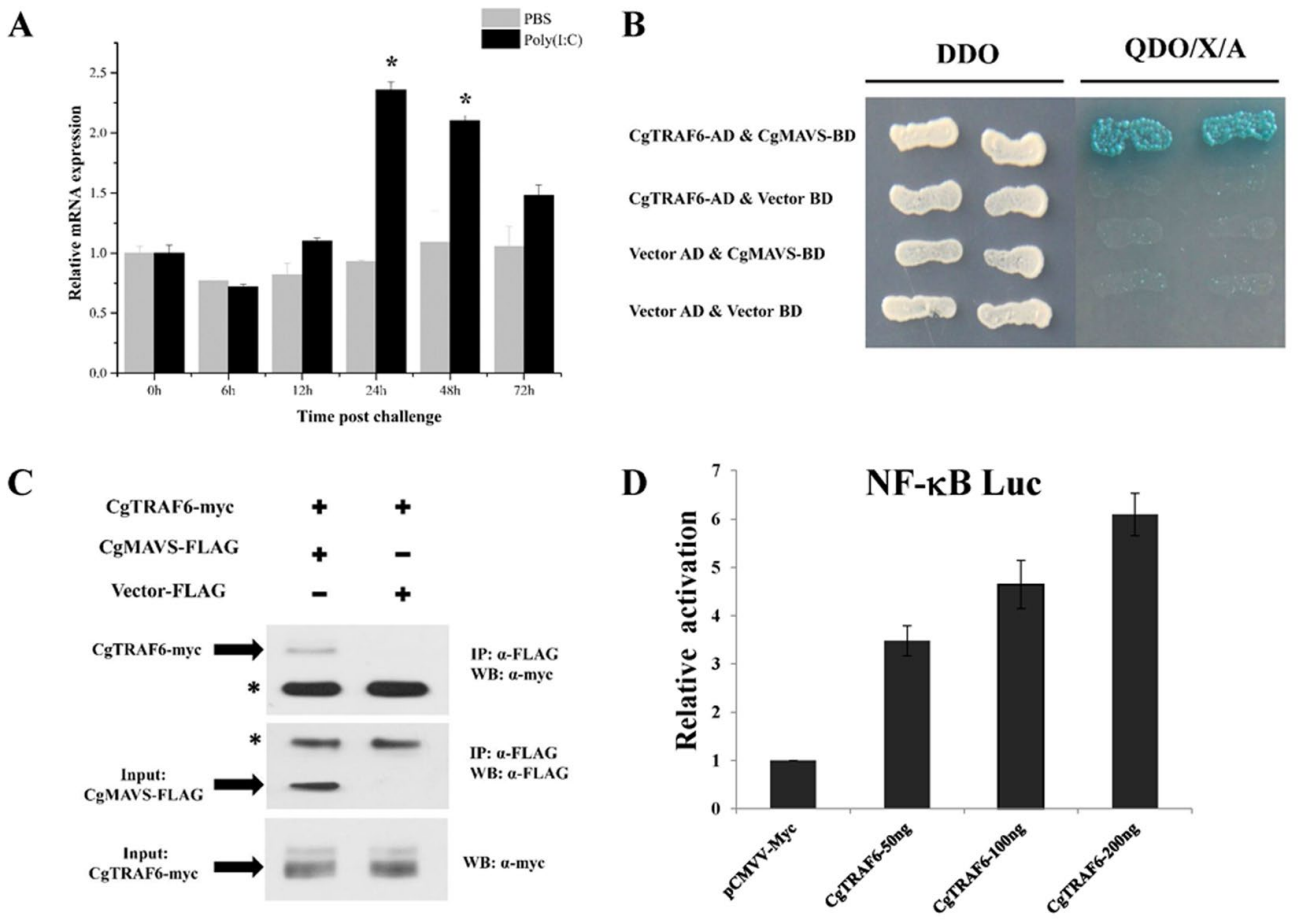
CgTRAF6 interacts with CgMAVS and specifically activates the NF-κB pathway. (**A**) Expression profile of *CgTRAF6* after poly(I:C) challenge determined by qRT-PCR. (**B** and **C**) Interaction between CgTRAF6 and CgMAVS detected by Y2H (**B**) and Co-IP (**C**). DDO: -Leu/-Trp double-dropout media, QDO/X/A: -Ade/-His/-Leu/-Trp quadruple-dropout media with X-α-Gal and aureobasidin. (**D**) CgTRAF6 specifically activated the NF-κB promoter in a concentration-dependent manner. The activation was detected using dual luciferase reporter assays in human HEK293T cells.

The RLR signalling adaptor MAVS needs to recruit other molecules such as TRAF6 to accomplish signal transduction^12, 14, 46^. Herein, we cloned the oyster *TRAF6* gene homolog, *CgTRAF6*. Analysis of the expression pattern of *CgTRAF6* mRNA after OsHV-1 challenge in oyster spat^26^ and larvae (Fig. 1C) showed only a slight upregulation in response to challenge. *CgTRAF6* expression was also slightly upregulated after poly(I:C) challenge (Fig. 6A). Y2H and co-IP assays showed that CgTRAF6 interacts with CgMAVS (Fig. 6B and C), suggesting that CgTRAF6 could be recruited by CgMAVS during oyster immune responses. Next, dual-luciferase reporter assays were employed to determine whether CgTRAF6 could activate transcription through the NF-κB, interferon-stimulated response element (ISRE), and interferon β (IFNβ) promoters. Overexpression of CgTRAF6 specifically activated NF-κB in a dose-dependent manner (Fig. 6D), but had no effect on the ISRE and IFNβ promoters (data not shown). This indicates that CgMAVS may specifically recruit CgTRAF6 during NF-κB activation. Nevertheless, CgMAVS itself could not activate the NF-κB promoter, and even attenuates CgTRAF6-dependent NF-κB activation in HEK293T cells (Supplementary Fig. 5).

### CgIRFs respond to virus challenge and activate the IFNβ and ISRE promoters

**Figure 7:**
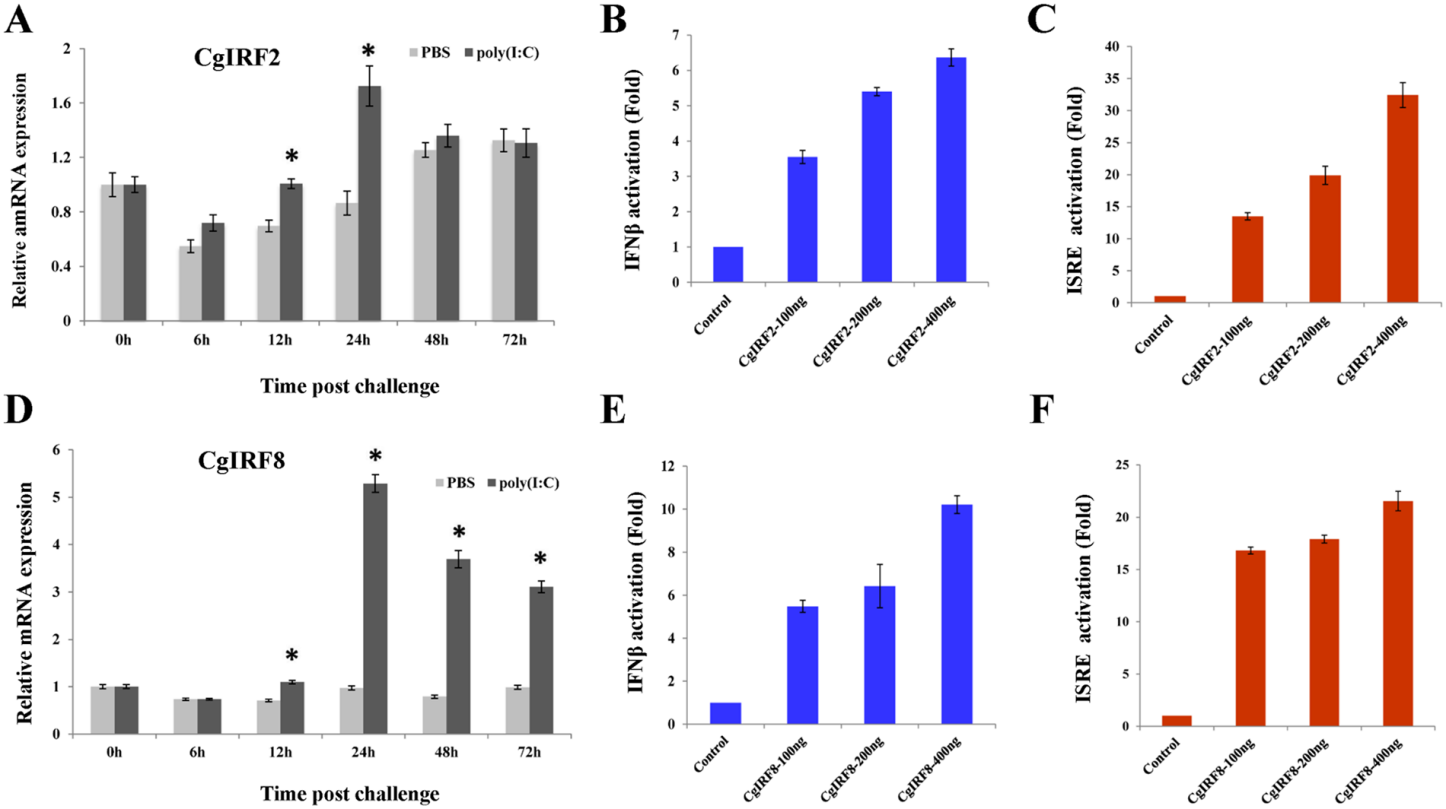
CgIRF2 and CgIRF8 respond to poly(I:C) challenge and activate human IFNβ and ISRE promoters. (**A**) Expression profile of *CgIRF2* mRNA after poly(I:C) challenge analysed by qRT-PCR. (**B** and **C**) CgIRF2 specifically activated the human IFNβ (**B**) and ISRE (**C**) promoters in a concentration-dependent manner. The activation was detected using dual luciferase reporter assays in human HEK293T cells. (**D**) Expression profile of *CgIRF8* mRNA after poly(I:C) challenge analysed by qRT-PCR. (**E** and **F**) CgIRF8 specifically activated the human IFNβ (**E**) and ISRE (**F**) promoters in a concentration-dependent manner.

In addition to its interaction with TRAF6, MAVS activates another signalling cascade, leading to the phosphorylation and activation of the transcription factors IRF3 and IRF7, which in turn activate the expression of type I *IFN* genes and responses to virus infection^18^. In the present study, two oyster *IRF* genes were identified and named *CgIRF2* and *CgIRF8*, as their sequences are most similar to the vertebrate *IRF2* and *IRF8* genes. The expression of *CgIRF2* and *CgIRF8* mRNA in oyster spat^26^ and larvae (Fig. 1D) showed dramatic upregulation after OsHV-1 challenge. Quantitative real-time PCR analysis revealed that *CgIRF2* and *CgIRF8* mRNA expression was induced by poly(I:C) challenge (Fig. 7A and D). Additionally, we investigated the mRNA expression patterns of *CgIRFs* after RNAi knockdown of *CgMAVS* to search for clues that oyster IRFs participate in the RLR signalling pathway. The qRT-PCR results showed that when *CgMAVS* expression was suppressed via RNAi (Supplementary Fig. 4), *CgIRF2* and *CgIRF8* mRNA expression decreased (Supplementary Figs 6 and 7). The similar expression profiles after poly(I:C) stimulation and RNAi suggest that CgIRFs and CgMAVS may function in the same signalling pathway, as suggested by Segal *et al*.^47, 48^.

To determine whether CgIRFs possess transcription factor activity similar to that of vertebrate IRFs, dual luciferase reporter gene assays were performed to investigate the effect of CgIRF2 and CgIRF8 on the IFNβ and ISRE promoters. The results showed that as the amount of transfected CgIRF2-expressing plasmid was increased from 100 to 200 and 400 ng, the activity of the IFNβ promoter was upregulated by 3.6, 5.4, and 6.4 fold, respectively, compared with the control (Fig. 7B). CgIRF2 also activated the ISRE promoter in a dose-dependent manner (Fig. 7C) and CgIRF8 similarly activated transcription from the IFNβ and ISRE promoters (Fig. 7E and F). These results suggest that CgIRFs may act as transcriptional regulators through promoters containing mammalian IFNβ and ISRE sequences. We also noticed that CgMAVS itself could not activate the IFNβ or ISRE promoter in human cells and the overexpressed CgMAVS attenuates the CgIRF2 or CgIRF8-dependent IFNβ and ISRE promoter activation (Supplementary Figs 8–11).

Finally, to directly assess the subcellular localization of CgIRF2 and CgIRF8, HeLa cells were transfected with plasmids encoding GFP-tagged CgIRF2 and CgIRF8, counterstained with Hoechst to identify nuclei, and examined by confocal laser-scanning microscopy. The fluorescent signals representing the CgIRF2 and CgIRF8 fusion proteins were distributed in a disperse manner, in both the cytoplasm and the nucleus (Supplementary Fig. 12).

## Discussion

Animals are constantly threatened by the invasion of various viruses and have evolved systems of immune defence to eliminate infective viruses from the body. Innate immunity is one of the first lines of defence in animals and relies primarily on the recognition of PAMPs via PRRs. In this report, we describe the first identification of a functional antiviral RLR signalling framework in the Pacific oyster based on the characterization of number of genes involved in the RLR pathway. We analysed the roles of these genes in antiviral immunity, revealing aspects of the *C. gigas* RLR signalling pathway.

Transcriptomic analysis of virus-infected Pacific oysters provides an opportunity to identify immune-related genes and pathways that participate in antiviral immunity. A previous transcriptomic study reported that expression of almost all oyster genes in the RLR pathway was induced in oyster spat after OsHV-1 challenge^26^. We analysed the transcriptomes of oyster larvae infected by OsHV-1 at different developmental stages, and found that oyster larvae mortality occurred at the U1–U3 stage and that OsHV-1 began to proliferate before the onset of mortality (Fig. 1A), suggesting that the cause of mortality is likely to be virus infection, rather than an effect of *Vibrio*. The results demonstrated that numerous genes of the RLR pathway were differentially regulated in response to viral challenge in oyster larvae (Fig. 1B–D). The expression profiles of the RLR genes investigated in our study were very similar, with expression peaking at the U3 stage. Interestingly, the expression patterns of genes encoding RLR proteins without CARD domains were identical to those containing CARD domains. The function of RLRs without CARD domains has not been well studied, although it is thought that they may function as modulators or suppressors of RLR signalling^49^. The expression profile of genes encoding RLR proteins without CARD domains identified in this study suggests that they may be involved in antiviral immunity in oyster larvae; however, determination of their function will require further study. The expression profiles of other signalling molecules were similar to those of RLR genes, except for *CgTRAF6*. The different expression profile of *CgTRAF6* may indicate that it is an important gene that regulates the development of *C. gigas* larvae, or that it is regulated by other factors.

The induced gene expression of RLR pathway genes after virus challenge make us speculate that there is a functional RLR signalling pathway in the Pacific oyster. Antiviral signalling is initiated by the recognition of PAMPs by PRRs. RLRs are crucial PRRs located in the cytoplasm that sense viral RNA PAMPs^50^. The RLR identified in this report, CgRIG-I-1, has functional domains similar to those of a previously identified oyster RLR^51^ and vertebrate RLRs with CARD domains^49^ (Fig. 2A). The expression of CgRIG-I-1 was induced by OsHV-1 and poly(I:C) challenge, suggesting that it could be involved in oyster antiviral immunity. Given that poly(I:C) can induce a protective antiviral immune response in oysters against subsequent viral challenge and that CgRIG-I-1 possesses a helicase domain similar to that of human RIG-I, which can bind poly(I:C) directly *in vitro* ^7^, we examined the interaction between poly(I:C) and CgRIG-I-1 and found that CgRIG-I-1 could bind poly(I:C) directly *in vitro* (Fig. 3C). These results indicate that CgRIG-I-1 may be a molecular PRR of oyster that recognizes virus PAMPs, acting as a crucial trigger for downstream signalling cascades. Considering that more than ten RLRs exist in the oyster genome^39^, the virus recognition mechanisms of oyster RLRs requires further investigation.

After sensing viral infections, RLRs interact with downstream adaptor proteins to initiate signalling cascades. MAVS is the crucial adaptor protein that binds RLRs via CARD-CARD interaction^12^; however, no MAVS homolog was previously characterized in invertebrates. In this study, we identified the first invertebrate *MAVS* gene in the Pacific oyster, *C. gigas*. Similar to vertebrate MAVS proteins, CgMAVS possesses an N-terminal CARD domain and C-terminal transmembrane domain (Fig. 2B). In contrast to vertebrate MAVS proteins, which include a proline-rich domain following the CARD domain, CgMAVS contains a Death domain. This difference may result in the recruitment of differing downstream signalling molecules by MAVS, suggesting differing signalling transduction patterns between oyster and vertebrate RLRs.

An RNAi assay demonstrated that knockdown of CgMAVS resulted a clear increase in mortality after OsHV-1 challenge (Fig. 4A), suggesting the important roles that CgMAVS plays in oyster antiviral immunity. To investigate the roles that MAVS protein plays in signal transduction, we generated a number of truncated mutant proteins to examine the relationship between CgRIG-I-1 and CgMAVS; however, no interaction was detected between full-length CgRIG-I-1 and CgMAVS in HEK293T cells. This may be due to the inactive state of CgRIG-I-1 in resting state cells^52^ or other unknown reasons. Next, we investigated the interactions between the CARD domains of CgRIG-I-1 and full-length CgMAVS. The results of Y2H and co-IP assays demonstrated an interaction between the CgRIG-I-1 CARD domains and CgMAVS, suggesting a conserved CARD-CARD interaction between CgRIG-I-1 and CgMAVS, and that oyster RIG-I-1 can recruit CgMAVS for signalling transduction. It has been reported that the C-terminal TM domain of MAVS is essential for its function^12^, and in our study, we found that CgMAVS lacking the TM domain failed to bind the CgRIG-I-1 CARD domains; however, when the TM domain of CgMAVS was replaced with that of human MAVS, the interaction between the CgRIG-I-1 CARDs and CgMAVS was rescued (Fig. 4C and D). These results indicate that the TM domain of CgMAVS is likely to be essential for its signalling and function. More extensive studies are required to determine the factors that influence the interaction between CgRIG-I-1 and CgMAVS. Additionally, the expression of *CgMAVS* itself was induced by challenge with poly(I:C) and OsHV-1, although the upregulation was not dramatic (Fig. 5A and B). Together, these data indicate that CgMAVS is likely to play an essential role in oyster antiviral responses and signal transduction.

On sensing a signal from RIG-I, MAVS dimerizes to create a signalling platform, leading to recruitment of members of the TRAF adaptor family and resulting in IRF3/7 activation mediated by TRAF3 or NF-κB activation mediated by TRAF2 and TRAF6^53^. MAVS TM-dependent self-association is crucial for mediation of antiviral immunity^16, 17^. The results of our study demonstrate that CgMAVS can form dimers and that the TM domain is essential for mediating this self-association (Fig. 5C and D). The dimerization of CgMAVS may be necessary for MAVS signalling, and more in-depth studies are required to determine the mechanisms regulating CgMAVS dimerization and elucidate the relationship between its dimerization and oyster antiviral immunity. As discussed above, some TRAF family members are signalling molecules recruited by MAVS, including TRAF2, TRAF3, and TRAF6. In our previous study, we did not detect interactions between CgMAVS and CgTRAF2^44^ or CgTRAF3 (data not shown). These differences in signalling transduction may be due to the different domain structure of CgMAVS, and it is possible that CgMAVS may recruit other TRAF molecules, as numerous TRAF family members are encoded in the oyster genome^39^. In this study, we identified a TRAF family member, CgTRAF6 that could interact with CgMAVS (Fig. 6B and C). The expression of *CgTRAF6* was induced slightly after poly(I:C) challenge (Fig. 6A). Importantly, CgTRAF6 could activate the NF-κB promoter in a concentration-dependent manner in mammalian cell lines (Fig. 6D). These results indicate that oyster MAVS can recruit TRAF6, and TRAF6 may further activate NF-κB. In our study, we found that CgMAVS itself could not activate the NF-κB, and even attenuates CgTRAF6-dependent NF-κB activation in HEK293T cells (Supplementary Fig. 5). This result may due to the low similarity and different domain structure between the oyster and human MAVS, oyster MAVS could not function totally as a key adaptor protein in human cell lines. And the details of the signal transduction and activation mechanism involved in this process require further investigation.

As mentioned above, mammalian MAVS can also recruit TRAF3 and thereby activate IRF3 or IRF7, a process crucial for the induction and expression of IFN genes^19, 20^. Although no IFN homologs have been identified in *C. gigas*, several key elements of the IFN pathway are present in the oyster genome, including genes involved in JAK/STAT signalling, IRFs, and many ISGs; therefore, it has been hypothesized that the role of IFNs in the oyster may be assumed by genes without recognizable homology to vertebrate IFN^26^. Homologs of IRF3 and IRF7 were not found in the Pacific oyster genome. Rather, four IRF-like genes were identified: three are homologs of *IRF2* (*Cg05133*, *Cg21171* and *Cg21170*) and one is related to *IRF8* (*Cg03270*). In this study, two *IRF* genes, *CgIRF2* (*Cg21171*) and *CgIRF8* (*Cg03270*), were identified. The expression of *CgIRF2* and *CgIRF8* was induced after OsHV-1 challenge in oyster spat^26^ and larvae (Fig. 1D) and after poly(I:C) challenge (Fig. 7A and D). Importantly, CgIRF2 and CgIRF8 appear to be able to regulate transcription through promoters containing mammalian IFNβ and ISRE sequences, though CgMAVS have no effect on the IFNβ and ISRE promoter. The subcellular localization of CgIRF2 and CgIRF8 in HeLa cells indicates that they may function in both the cytoplasm and the nucleus. In summary, these results suggest that oyster IRF genes may be crucial transcription factors with conserved functions in antiviral immunity. As oysters possess only four IRF genes, whereas there are nine such genes in mammals with various functions^54^, functional diversification may have occurred during IRF gene evolution. Oyster IRFs may have functions similar to those of vertebrate IRF3 or IRF7, participating in antiviral signalling. Interestingly, the similar expression profiles observed after poly(I:C) stimulation and MAVS-RNAi knockdown suggest that CgIRFs and CgMAVS may function in the same signalling pathway^47^. However, the signal transduction from CgMAVS to CgIRFs and mechanisms underlying activation of oyster IRFs require additional in-depth research.

In conclusion, we demonstrate for the first time that a MAVS-dependent RLR antiviral signalling pathway is conserved and functional in the Pacific oyster. AS a PRR, CgRIG-I-1 could bind poly(I:C) directly *in vitro*. CgMAVS is the first cloned invertebrate MAVS gene. Similar to human MAVS, CgMAVS interact with CgRIG-I-1 by a CARD-CARD interaction and could form homodimers in TM-dependent manner; CgMAVS also recruits CgTRAF6, and CgTRAF6 subsequently activates NF-κB. However, unlike human MAVS, CgMAVS could not interact with CgTRAF3, suggesting differing signalling transduction patterns between oyster and vertebrate RLRs. Additionally, oyster IRFs might function downstream of CgMAVS and were able to activate the IFNβ and ISRE promoter in mammalian cells. Our study will not only contribute to the understanding of antiviral mechanisms in invertebrates and the evolution of the vertebrate RLR signalling pathway, but also provide resources for further investigations into oyster immunity, and make contribution to the finding antiviral strategies for virus control in oysters.

## Materials and Methods

### Ethics statement

Experiments in this study were conducted with approval from Experimental Animal Ethics Committee, Institute of Oceanology, Chinese Academy of Sciences, China.

### Animals and pathogens

Healthy Pacific oysters, with an average shell height of 60 mm, were collected from a farm in Qingdao, Shandong Province, China. All animal experiments were conducted in accordance with the guidelines and approval of the respective Animal Research and Ethics Committees of the Chinese Academy of Sciences. Experimental specimens were acclimatized in aerated and filtered seawater at 22 ± 0.5 °C for more than one week prior to execution of experiments. OsHV-1 suspension was prepared from the gill and mantle tissues of virus-infected oysters and stored at −80 °C according to the previously published methods^55^.

### Cloning and sequence analysis of oyster RLR pathway genes

Total RNA was extracted from oyster gill and mantle samples using TRIzol Reagent (Invitrogen, USA) and then treated with DNase I (Promega, USA). First-strand cDNA synthesis using the treated RNA as a template was performed using Promega M-MLV reverse transcriptase according to the manufacturer’s instructions. Then using information from the *C. gigas* genome^38^, gene fragments were amplified using specific primers (Supplementary Table 1). Using sequence information from the amplified gene fragments, gene-specific primers were designed for 3′ and 5′ rapid amplification of cDNA ends (RACE). RACE cloning was performed using two rounds of nested PCR and the touchdown program according to previously described methods^44^. Finally, PCR products were purified using the E.Z.N.A Gel Extraction Kit (OMEGA, USA) and cloned into the pMD19-T vector (TaKaRa, Japan) and the recombinant vectors were transformed into Trans1-T1 competent cells (Transgen, China) and sequenced.

Open Reading Frame Finder (http://www.ncbi.nlm.nih.gov/gorf/orfig.cgi) was used to analyse cDNA sequences and deduce the corresponding polypeptides they encode. SMART (Simple Modular Architecture Research Tool) (http://smart.emblheidelberg.de) was used to predict protein domains. Protein sequences from different species were downloaded from NCBI (http://www.ncbi.nlm.nih.gov/guide/proteins/) and compared using the ClustalW2 program (http://www.ebi.ac.uk/Tools/msa/clustalw2/). A phylogenetic tree was constructed using the program MEGA (Version 5.05), with the neighbour-joining algorithm. Reliability of the branching was tested using bootstrap resampling (1000 pseudo-replicates) (http://www.megasoftware.net).

### Transcriptomic analysis of RLR genes in oyster larvae

Transcriptome data from oyster larvae infected with OsHV-1 were downloaded from the NCBI database, under the BioProject number PRJNA146329^38^. In addition, transcriptome data from normally developed oysters not infected with virus were also downloaded from the NCBI database, under the BioProject number PRJNA277901^38^. The gene expression data measured by RPKM (reads per kilobase per million mapped reads) were acquired from these two published data.

### Expression analysis of RLR pathway genes

To analyse the expression profile of genes after challenge, *C. gigas* were divided into 3 groups (n = 50 per group), in which oysters received muscle injections with PBS (controls), 100 μg of poly(I:C), or 100 μL of OsHV-1 suspension at 10^4^ copies of viral DNA copies/μL. The haemolymph from challenged oysters was sampled at 0, 6, 12, 24, 48, and 72 h post injection, with each sample consisting of pooled haemolymph from six oysters. Samples were subjected to RNA extraction and qRT-PCR analysis. Briefly, qRT-PCR was performed in a 7500 Fast Real-Time PCR System (ABI, USA) using a SYBR Green Real Time PCR Master Mix kit (TaKaRa) to quantify the mRNA expression of genes. The primers used for the qRT-PCR analysis are listed in Supplementary Table 1. Glyceraldehyde-3-phosphate dehydrogenase (*GAPDH*) and β-actin (*ACTB*) genes were employed as internal controls for cDNA normalization.

### Poly(I:C) pull-down assay

We performed an *in vitro* poly(I:C) pull-down assay to detect the interaction between this synthetic dsRNA viral analogue poly(I:C) and oyster RIG-I-1 protein. Poly(I:C) was conjugated with biotin using an EZ-Link Psoralen-PEG3-Biotin kit (Thermo, USA) by exposure to UV (365 nm wavelength), according to the manufacturer’s instructions. Lysates from 293 cells transfected with the indicated plasmids were incubated with biotinylated-poly(I:C) for 1 h at room temperature and then incubated with streptavidin beads for a further 45 min at 4 °C. The beads were washed three times with lysis buffer and analysed by immunoblotting with an anti-Myc antibody (Roche, USA).

### *CgMAVS* RNAi assays

Small interfering RNAs (siRNAs) targeting *CgMAVS* were synthesized by GenePharma (Shanghai, China) based on the gene sequence (Supplementary Table 1) along with a negative control (NTC) siRNA, consisting of a scrambled version of the *CgMAVS*-targeting sequence. Then, 150 oysters were randomly divided into three groups of 50 individuals each. In the first group, each oyster received a muscle injection of 100 mg CgMAVS-siRNA, whereas the control groups received injections of NTC-siRNA (100 mg per oyster) or PBS. At 0, 24, 48, 72, 96, 120, 144, and 168 h after injection, three individuals from each group were randomly chosen for haemolymph collection. These samples were immediately centrifuged at 800 *g* for 10 min at 4 °C to harvest the haemocytes for RNA extraction. The qRT-PCR template was prepared and qRT-PCR was performed to determine mRNA expression levels of *CgMAVS* or downstream genes following knockdown of *CgMAVS*. For virus challenge tests, healthy oysters were injected with siRNA and, 72 h later, challenged with 10^6^ viral DNA copies suspended in 100 μL of PBS. Oysters were maintained in culture flasks following infection. Experiments were performed in triplicate and cumulative mortality was recorded every 24 h after virus injection.

### Plasmid construction, cell culture, and transfection

The open reading frame (ORF) regions of each gene were amplified using Phusion High-Fidelity DNA polymerase (Thermo) with specific primers (Supplementary Table 1). pCMV-Myc (Clontech, USA), pEGFP-N1 (Clontech), and pCMS-EGFP-FLAG plasmids (constructed by our lab) were digested with EcoRI, XhoI, and XhoI (New England Biolabs, USA), respectively, and the purified PCR products were fused with the purified digested plasmids using the Ligation-Free Cloning System (Applied Biological Materials, Inc., Canada), according to the manufacturer’s instructions.

HeLa cells (ATCC, USA) were cultured in modified Roswell Park Memorial Institute (RPMI)-1640 medium (HyClone, USA), and HEK293T cells (ATCC) were cultured in Dulbecco’s modified Eagle medium (high glucose) (HyClone). Both types of media were supplemented with 10% heat-inactivated foetal bovine serum (Gibco, USA) and 1× penicillin-streptomycin solution (Solarbio, China). Cells were grown in an atmosphere of 95% air/5% CO_2_ at 37 °C and subcultured every 3–4 days. Plasmids were transfected into HeLa or HEK293T cells using Lipofectamine 3000 reagent (Life Technologies, USA) according to the manufacturer’s instructions.

### Yeast two-hybrid assay

Yeast two-hybrid (Y2H) assays were performed to detect interactions between proteins. Briefly, using the Y2H system (Clontech Matchmaker Gold Yeast Two-Hybrid System; TaKaRa), the fusion protein expression plasmids pGADT7 (AD vector) and pGBKT7 (BD vector) were transformed into the Y187 and Gold yeast strains, respectively, according to the manufacturer’s instructions. Y187 cells were cultured on selective plates with synthetically defined (SD) medium lacking leucine (SD/-Leu), whereas Gold cells were cultured on SD plates lacking tryptophan (SD/-Trp). After 3–5 days, yeast strains able to grow on SD/-Leu and SD/-Trp medium were hybridized in 2× yeast extract peptone dextrose (YPDA) medium and selected on double drop-out (SD/-Leu/-Trp) medium. Interactions between proteins were detected based on the ability of the hybridized clones to grow on quadruple drop-out (SD/-Ade/-His/-Leu/-Trp) medium supplemented with X-α-Gal and aureobasidin A (TaKaRa).

### Co-immunoprecipitation assays

HEK293T cells were divided between two or more Petri dishes (10 cm diameter, Corning, USA) and cultured for 24 h. Fused pCMV-Myc plasmids were co-transfected with vectors expressing FLAG-tagged fusion proteins or empty FLAG vector (control). After 24 h, cells were harvested in cell lysis buffer (Beyotime). Input samples were prepared from the cell lysate and the remaining lysates were mixed with the anti-FLAG M2 magnetic beads (Sigma, USA) under gentle shaking on a roller at 4 °C for 2–4 h. The beads were then washed three times with cell lysis buffer. Input and co-IP samples were incubated with 2 × protein sodium dodecyl sulphate polyacrylamide gel electrophoresis loading buffer (TaKaRa) at 100 °C for 3–5 min. Proteins were analysed by western blotting using anti-Myc antibody and anti-FLAG antibodies (Sigma).

### Dual-luciferase reporter assays

Dual-luciferase reporter assays were performed in HEK293T cells to detect the effects of oyster proteins on transcription from the NF-κB, ISRE, and IFNβ promoters using Myc-fused protein expressing vectors. Briefly, cells in 24-well plates (Corning, USA) were transfected with 0.1 μg of reporter gene plasmids, 0.01 μg of pRL-CMV *Renilla* luciferase plasmid (Promega), and varying amounts of expression plasmids or empty expression vectors (as controls). The pRL-CMV *Renilla* luciferase plasmid was used as an internal control. At 24–48 h post transfection, the Dual-Luciferase Reporter Assays System (Promega) was used to measure the activity of firefly and *Renilla* luciferase according to the manufacturer’s instructions. Experiments were performed in triplicate.

### Subcellular localization assay

HeLa cells were transfected with pEGFP-CgIRF2, pEGFP-CgIRF8, or pEGFP-N1 and washed once with PBS 24 h post-transfection, followed by staining with Hoechst 33342 (Invitrogen, USA) in PBS (2 mg/mL). After staining for 10 min at 37 °C, cells were washed twice with PBS and then visualized using laser scanning confocal microscopy (Carl Zeiss, Germany).

## Electronic supplementary material

Supplemental Files
