# Supplementary material for: Characterization of the Mollusc RIG-I/MAVS Pathway Reveals an Archaic Antiviral Signalling Framework in Invertebrates
Source: Sci Rep. 2017 Aug 15;7:8217. doi: 10.1038/s41598-017-08566-x (PMC5557890; doi:10.1038/s41598-017-08566-x)
Supplement: Supplementary file 1 — Supplemental Files [file 41598_2017_8566_MOESM1_ESM.pdf]

Characterization of the Mollusc RIG-I/MAVS Pathway Reveals an Archaic Antiviral Signalling Framework in Invertebrates

Baoyu Huang[abd#], Linlin Zhang[abd#], Yishuai Du[abd], Fei Xu[abd], Li Li[acd*], Guofan Zhang[abd*]

Affiliations and addresses

[a] Key Laboratory of Experimental Marine Biology, Institute of Oceanology, Chinese Academy of Sciences, Qingdao, China 266071

[b] Laboratory for Marine Biology and Biotechnology, Qingdao National Laboratory for Marine Science and Technology, Qingdao, China 266071

[c] Laboratory for Marine Fisheries and Aquaculture, Qingdao National Laboratory for Marine Science and Technology, Qingdao, China 266071

[d] National & Local Joint Engineering Laboratory of Ecological Mariculture, Institute of Oceanology, Chinese Academy of Sciences, Qingdao, China 266071

[#] These authors contributed equally to this work.

[*]Correspondence to: Dr. Guofan Zhang
Institute of Oceanology, Chinese Academy of Sciences
7th Nanhai Rd., Qingdao, China
Tel.: +86-532-82896728; fax: +86-532-82896728.
E-mail: gzhang@qdio.ac.cn (Guofan Zhang)
*Correspondence to: Dr. Li Li
Institute of Oceanology, Chinese Academy of Sciences
7th Nanhai Rd., Qingdao, China
Tel.: +86-532-82896727; fax: +86-532-82896727.
E-mail: lili@qdio.ac.cn (Li Li)

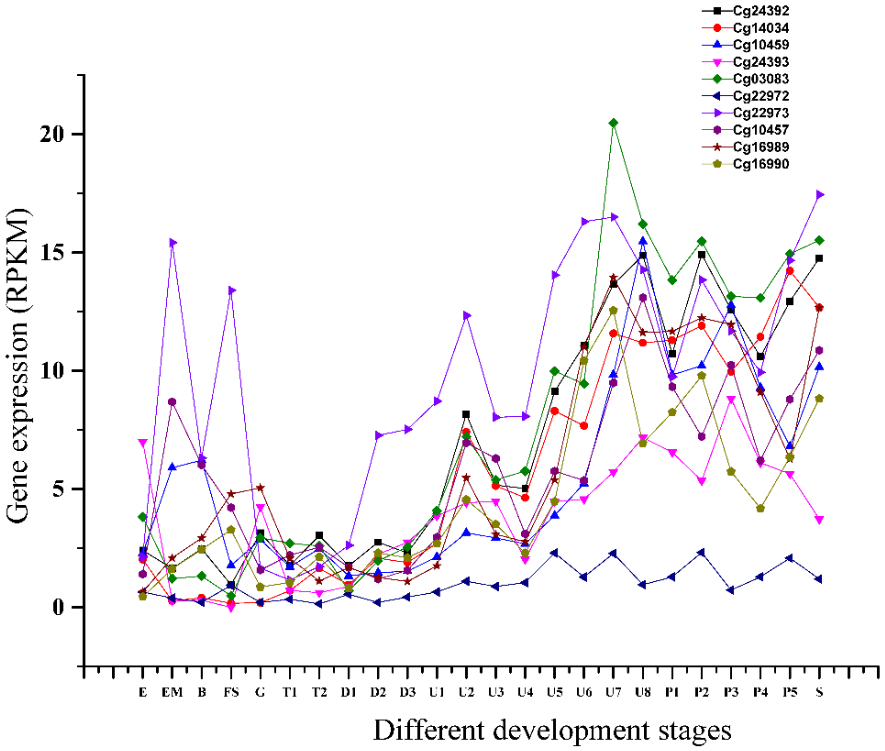

Supplementary Figure 1 Expression of RIG-I like receptors (RLRs) in oyster larvae. *Cg24392*, *Cg14034*, *Cg22973*, and *Cg03083* are RLRs with CARD domains; the left genes are RLRs without CARD domains. Times of sampling of oyster larvae and abbreviations for developmental stages are the same as those in Figure 1. The abbreviations are: E: Egg; TC: Two cells; FC: Four cells; EM: Early morula; M: Morula; B: Blastula; RM: Rotary movement; FS: Free swimming; EG: Early gastrula; G: Gastrula; T: Trochophore; ED: Early D-shape larvae; D: D-shape larvae; EU: Early umbo larva; U: Umbo larva; LU: Later umbo larva; P: Pediveliger. S: Spat.

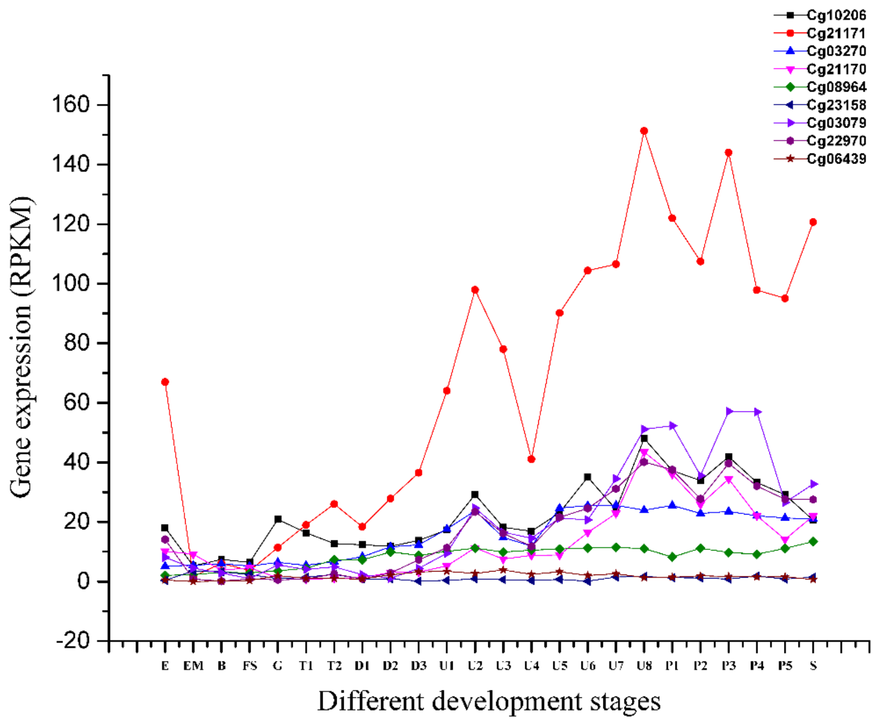

Supplementary Figure 2 Expression of genes involved in the RLR signaling pathway, including *TRAF2* (*Cg08964*), *TRAF3* (*Cg23158*), *TRAF6* (*Cg10206*), three *MITA* (transmembrane protein 173) genes (*Cg06439*, *Cg03079* and *Cg22970*) and three *IRF* genes (*Cg21170*, *Cg21171*, and *Cg03270*). Times of sampling of oyster larvae and abbreviations for developmental stages are the same as those in Figure 1. The abbreviations are: E: Egg; TC: Two cells; FC: Four cells; EM: Early morula; M: Morula; B: Blastula; RM: Rotary movement; FS: Free swimming; EG: Early gastrula; G: Gastrula; T: Trochophore; ED: Early D-shape larvae; D: D-shape larvae; EU: Early umbo larva; U: Umbo larva; LU: Later umbo larva; P: Pediveliger. S: Spat.

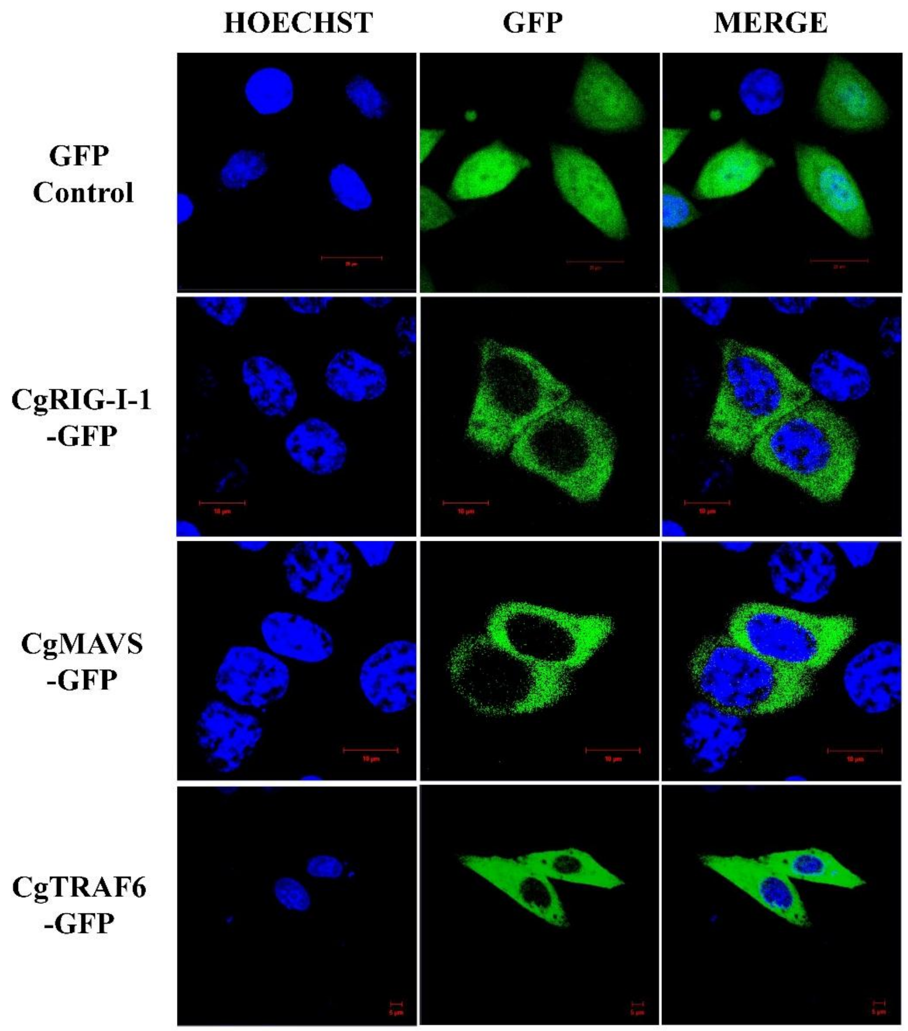

Supplementary Figure 3 Subcellular localization of CgRIG-I-1, CgMAVS and CgTRAF6 in HeLa cells. The left-hand panels depict Hoechst staining, the middle panels depict GFP staining, and the right-hand panels depict merged Hoechst/GFP staining. The upper panels depict localization of the GFP negative control, and the lower three panels depict localization of the CgRIG-I-1, CgMAVS and CgTRAF6 protein.

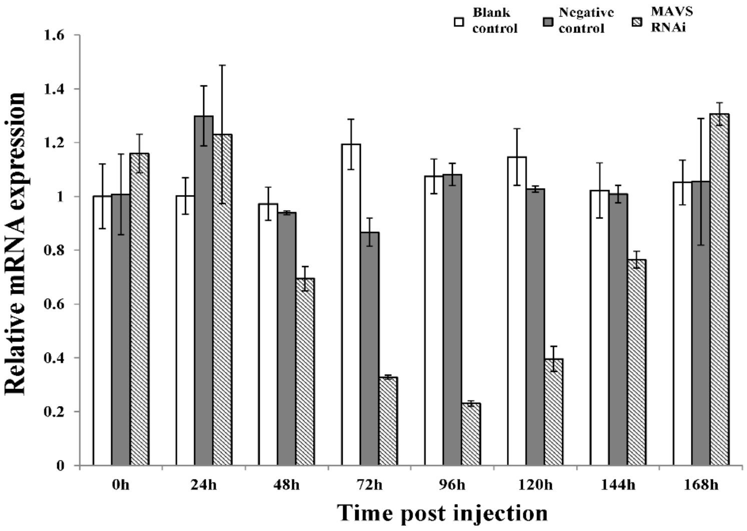

Supplementary Figure 4 The expression profile of *CgMAVS* mRNA after RNAi. Elongation factor (EF) gene expression was used as an internal control and time 0 h was used as a reference sample. Vertical bars represent the mean ± SD (N = 3).

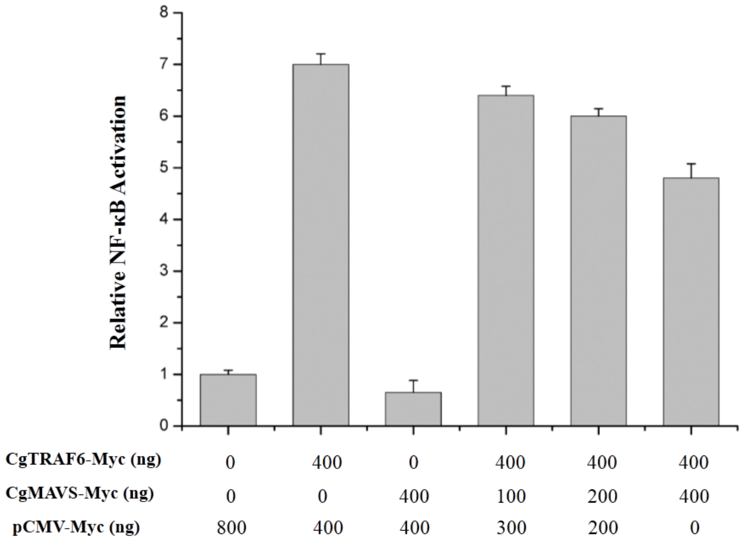

Supplementary Figure 5 The oyster MAVS protein attenuates the NF-κB activation by CgTRAF6 in a dose-dependent manner. Cells were transiently co-transfected with an NF-kB-dependent luciferase reporter vector and pRL-CMV, together with CgTRAF6 and CgMAVS expression vector. The expression vector pCMV-Myc was used as controls. Luciferase activities were normalized to Renilla luciferase activity and expressed as the fold stimulation relative to that measured in cells transfected with the empty vector.

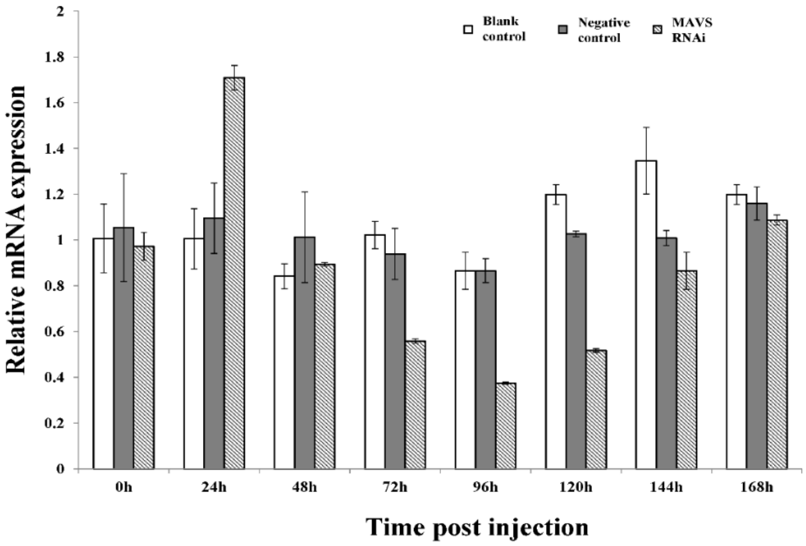

Supplementary Figure 6 The expression profile of *CgIRF2* mRNA after *CgMAVS*-RNAi. Elongation factor (EF) gene expression was used as an internal control and time 0 h was used as a reference sample. Vertical bars represent the mean ± SD (N = 3).

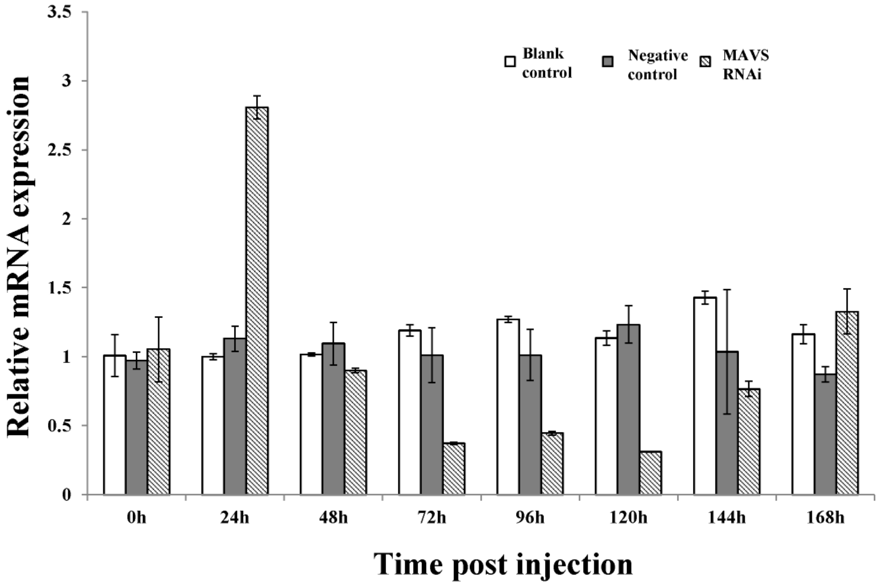

Supplementary Figure 7 The expression profile of *CgIRF8* mRNA after *CgMAVS*-RNAi. Elongation factor (EF) gene expression was used as an internal control and time 0 h was used as a reference sample. Vertical bars represent the mean ± SD (N = 3).

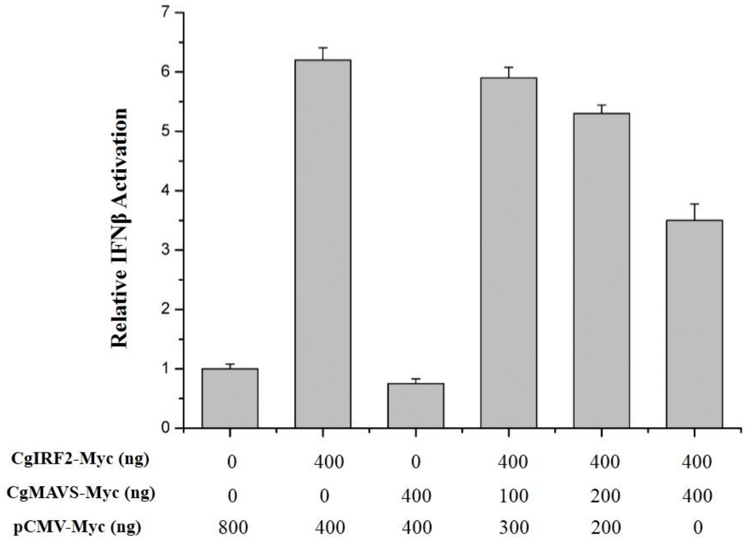

Supplementary Figure 8 The oyster MAVS protein attenuates the human IFNβ activation by CgIRF2 in a dose-dependent manner. Cells were transiently co-transfected with human IFNβ luciferase reporter vector and pRL-CMV, together with CgIRF2 and CgMAVS expression vector. The expression vector pCMV-Myc was used as controls. Luciferase activities were normalized to Renilla luciferase activity and expressed as the fold stimulation relative to that measured in cells transfected with the empty vector.

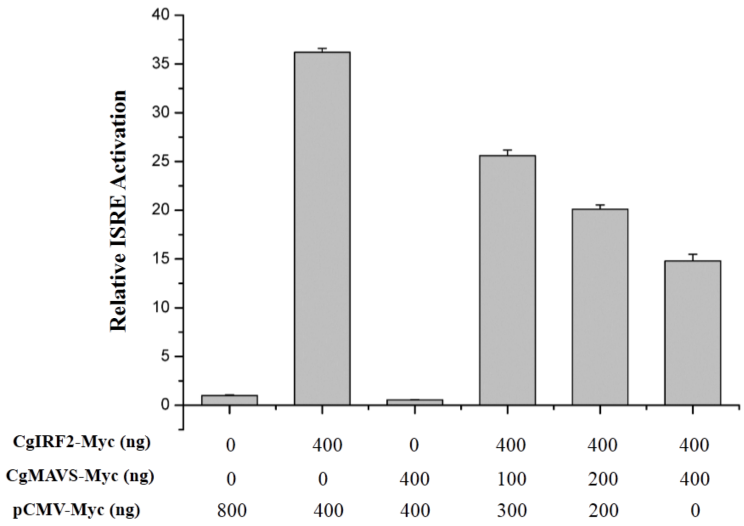

Supplementary Figure 9 The oyster MAVS protein attenuates the ISRE-containing luciferase reporter activation by CgIRF2 in a dose-dependent manner. Cells were transiently co-transfected with an ISRE-containing luciferase reporter vector and pRL-CMV, together with CgIRF2 and CgMAVS expression vector. The expression vector pCMV-Myc was used as controls. Luciferase activities were normalized to Renilla luciferase activity and expressed as the fold stimulation relative to that measured in cells transfected with the empty vector.

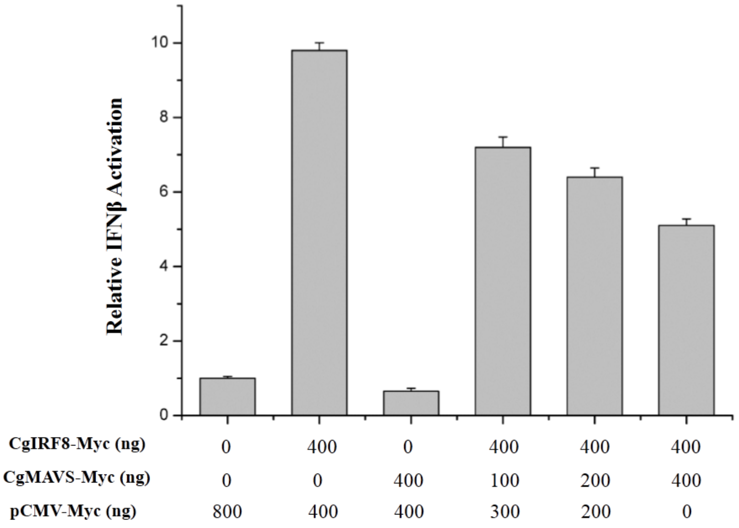

Supplementary Figure 10 The oyster MAVS protein attenuates the human IFNβ activation by CgIRF8 in a dose-dependent manner. Cells were transiently co-transfected with human IFNβ luciferase reporter vector and pRL-CMV, together with CgIRF8 and CgMAVS expression vector. The expression vector pCMV-Myc was used as controls. Luciferase activities were normalized to Renilla luciferase activity and expressed as the fold stimulation relative to that measured in cells transfected with the empty vector.

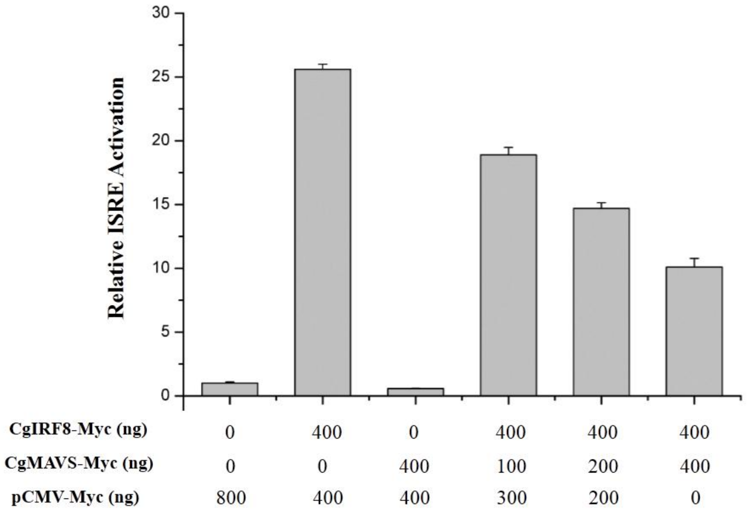

Supplementary Figure 11 The oyster MAVS protein attenuates the ISRE-containing luciferase reporter activation by CgIRF8 in a dose-dependent manner. Cells were transiently co-transfected with an ISRE-containing luciferase reporter vector and pRL-CMV, together with CgIRF8 and CgMAVS expression vector. The expression vector pCMV-Myc was used as controls. Luciferase activities were normalized to Renilla luciferase activity and expressed as the fold stimulation relative to that measured in cells transfected with the empty vector.

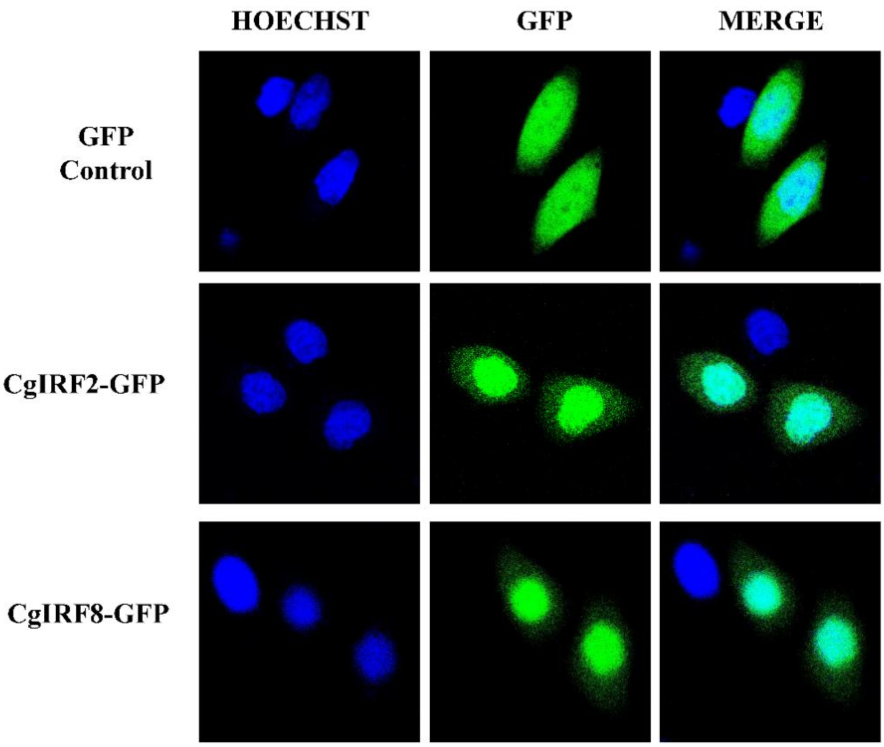

Supplementary Figure 12 Subcellular localization of CgIRF2 and CgIRF8 in HeLa cells. The left-hand panels depict Hoechst staining, the middle panels depict GFP staining, and the right-hand panels depict merged Hoechst/GFP staining. The upper panels depict localization of the GFP negative control, and the lower two panels depict localization of the CgIRF2-GFP and CgIRF8-GFP protein. The green fluorescent signal of CgIRFs-GFP was most strongly focused in the cytoplasm and nucleus.

Supplementary Table 1 Primers used in this research

Primer	Sequence（5’-3’）	Application
CgRIG-I-1-F	ATCAACCTCCGCAGTTCCATTAGC	Cloning
CgRIG-I-1-R	CCTTCCTCGGCCACAGATGTAGCA	Cloning
3CgRIG-I-1-1	GCATCCCTGCTAAACCTCTAAACT	3’ RACE
3CgRIG-I-1-2	GCGTTGGGACGAACACCA	3’ RACE
5CgRIG-I-1-1	GCAAGTTTAGAGGTTTAGCAGGGAT	5’ RACE
5CgRIG-I-1-2	TGGATGTTTGCCCTCAGATTTGTTG	5’ RACE
CgMAVS-F	ACGACTAATAATGCCATTCCAATC	Cloning
CgMAVS-R	ATATGCTTCCCAAACTGTCCACT	Cloning
3CgMAVS-1	TCACCTGATGAATTATTGCCCCAC	3’ RACE
3CgMAVS-2	AAGATGTCGGTTGGCACAAGAGGGT	3’ RACE
5CgMAVS-1	CCTGTTTGTGCAGCCAAATCTTTCC	5’ RACE
5CgMAVS-2	GGAGGCATCTTCTCATTCTGTGGTG	5’ RACE
CgTRAF6-F	AATGACGTCAGAGCGACCTTTG	Cloning
CgTRAF6-R	CAAAGGTCGCTCTGACGTCATT	Cloning
3CgTRAF6-1	GGCGAGGTAACGGCAATTCATA	3’ RACE
3CgTRAF6-2	AGTGGCCCTTTAGTGGTCGTAT	3’ RACE

5CgTRAF6-1	GTAAGGAATGGCTGTGGGTGAG	5’ RACE
5CgTRAF6-2	GTGGCAGATGTGGGATAGGTG	5’ RACE
CgIRF2-F	CGTGTGATTTAACAGGTGCAAT	Cloning
CgIRF2-R	TATAGTCAATGAGTGCCACTAGA	Cloning
3CgIRF2-1	TGATGGCGATGGACAGCGAAGG	3’ RACE
3CgIRF2-2	CCACCGACCTCACAGCCCTTCA	3’ RACE
5CgIRF2-1	CTTCGCTGTCCATCGCCATCA	5’ RACE
5CgIRF2-2	TGGCACTGTCGTTCGTTCTG	5’ RACE
CgIRF8-F	AGAGTTAGCCGTATCTTCTCG	Cloning
CgIRF8-R	GAAACAAGGGATCCACACTCTG	Cloning
dTAP	GGCCACGCGTCGACTAGTACT16	RACE
dGAP	GGCCACGCGTCGACTAGTACG10	RACE
AP	GGCCACGCGTCGACTAGTAC	RACE
CgRIG-I-1-QF	TCGTAGGACCTGAACATCCAATC	qRT-PCR
CgRIG-I-1-QR	TTTCCACTTCCAGTAGGAGCAAC	qRT-PCR
CgMAVS-QF	GCTTTATGTCATCCTGCTGTTGG	qRT-PCR
CgMAVS-QR	CTTCTCATTCTGTGGTGCTCCTTTA	qRT-PCR
CgTRAF6-QF	TCTGCCACAAAAGAACCCTCAT	qRT-PCR
CgTRFA6-QR	TGGACCGCCGCATACAAT	qRT-PCR
CgIRF2-QF	CTTGTTATGAGGAGGGACTGG	qRT-PCR
CgIRF2-QR	GGTACTTTTGAAGGGCTGTGAG	qRT-PCR
CgIRF8-QF	CTTTCTCGTGGGATTTGGTC	qRT-PCR
CgIRF8-QR	CGATGTTTGGCTCTGCTATG	qRT-PCR
EF- qRT-F	AGTCACCAAGGCTGCACAGAAAG	qRT-PCR
EF- qRT-R	TCCGACGTATTTCTTTGCGATGT	qRT-PCR
β-actin-F	GTGCTACGTTGCCCTGGACTT	qRT-PCR
β-actin-R	TCGCTCGTTGCCAATGGTGAT	qRT-PCR
MAVS-siRNA	GUGAUGUUUCAAACAGAGAAG	MAVS-RNAi
NTC-siRNA	UGGUAAGAACGUGUAACGUAA	MAVS-RNAi
CgRIG-I-1_CARD-myc-R	CTCGGTCGACCGAATTTCAATCACCAAGGG TATTTTGGGGA	Protein expression
CgRIG-I-1 -myc-F	CATGGAGGCCCGAATTATGGGGTTAGCTAAC GGCCCAGTCAGT	Protein expression
CgRIG-I-1 -myc-R	CTCGGTCGACCGAATTTTACATCATATCAAT GAGTTTCTCTCCAC	Protein expression
CgMAVS-myc-F	CATGGAGGCCCGAATTATGGGGTCAATTGA AGATGACTATC	Protein expression
CgMAVS-myc-R	CTCGGTCGACCGAATTCTATCTGAAAACGC AGCGATGAAAAATAAAAG	Protein expression
CgTRAF6-myc-F	CATGGAGGCCCGAATTATGGGGACGTCAGA GCGACCTTTG	Protein expression
CgTRAF6-myc-R	CTCGGTCGACCGAATTTCACTCCGATCTCCC AATGATCTGA	Protein expression
CgIRF-2-myc-F	CATGGAGGCCCGAATTATGGTGCAGAGAAA GCAAAGTCAGC	Protein expression
CgIRF-2-myc-R	CTCGGTCGACCGAATTTCATAACTGGGACAT GCGAAGACC	Protein expression
CgIRF-8-myc-F	CATGGAGGCCCGAATTATGGCAACAGAAAT TGATATTCGCAG	Protein expression
CgIRF-8-myc-R	CTCGGTCGACCGAATTTCAGGTTTCCATTTG	Protein expression

	TCCATGGCAT	
CgMAVS-FLAG-F	CTCCATATGACTAGTCTCGAGATGGGGTCAATTGAAGATGACTATC	Protein expression
CgMAVS-FLAG-R	TACCACGCGTGAATTCTCGAGCTATCTGAAAACGCAGCGATGAAAAATAAAAG	Protein expression
CgMAVS-HsTM-FLAG-F	CTCCATATGACTAGTCTCGAGATGGGGTCAATTGAAGATGACT	Protein expression
CgMAVS-HsTM-FLAG-R	TACCACGCGTGAATTCTCGAGCTAGTGCAGACGCCGCCG	Protein expression
CgMAVS-ΔTM-FLAG-F	CTCCATATGACTAGTCTCGAGATGGGGTCAATTGAAGATGACT	Protein expression
CgMAVS-ΔTM-FLAG-R	TACCACGCGTGAATTCTCGAGTCATGTACATAATTTCTTGTTCGAA	Protein expression
CgRIG-I-1-GFP-F	GGACTCAGATCTCGAGATGGGGTTAGCTAACGGCCCAGTCAGT	Subcellular localization
CgRIG-I-1-GFP-R	GAAGCTTGAGCTCGAGCATCATATCAATGAGTTTCTCTCCAC	Subcellular localization
CgMAVS-GFP-F	GGACTCAGATCTCGAGATGGGGTCAATTGAAGATGACTATC	Subcellular localization
CgMAVS-GFP-R	GAAGCTTGAGCTCGAGTCTGAAAACGCAGCGATGAAAAATAAAAG	Subcellular localization
CgTRAF6-GFP-F	GGACTCAGATCTCGAGATGGGGACGTCAGAGCGACCTTTG	Subcellular localization
CgTRAF6-GFP-R	GAAGCTTGAGCTCGAGCTCCGATCTCCCAATGATCTGA	Subcellular localization
CgIRF2-GFP-F	GGACTCAGATCTCGAGATGGTGCAGAGAAAGCAAAGTCAGC	Subcellular localization
CgIRF2-GFP-R	GAAGCTTGAGCTCGAGTAACTGGGACATGCGAAGACC	Subcellular localization
CgIRF8-GFP-F	GGACTCAGATCTCGAGATGGCAACAGAAATTGATATTCGCAG	Subcellular localization
CgIRF8-GFP-R	GAAGCTTGAGCTCGAGGGTTTCCATTTGTCCATGGCAT	Subcellular localization
CgRIGI-1_CARD-AD-F	GGAGGCCAGTGAATTCATGGGGTTAGCTAACGGCCC	Y2H
CgRIGI-1_CARD-AD-R	CGAGCTCGATGGATCCTCAATCACCAAGGGTATTTTGGGGA	Y2H
CgMAVS-AD-F	GGAGGCCAGTGAATTCATGGGGTCAATTGAAGATGACTATC	Y2H
CgMAVS-AD-R	CGAGCTCGATGGATCCCTATCTGAAAACGCAGCGATGAAAAATAAAAG	Y2H
CgMAVS-ΔTM-AD-F	GGAGGCCAGTGAATTCATGGGGTCAATTGAAGATGACT	Y2H
CgMAVS-ΔTM-AD-R	CGAGCTCGATGGATCCTCATGTACATAATTTCTTGTTCGAA	Y2H
CgMAVS-BD-F	CATGGAGGCCGAATTCATGGGGTCAATTGAAGATGACTATC	Y2H
CgMAVS-BD-R	GCAGGTCGACGGATCCCTATCTGAAAACGCAGCGATGAAAAATAAAAG	Y2H
CgMAVS-ΔTM-BD-F	CATGGAGGCCGAATTCATGGGGTCAATTGA	Y2H

CgMAVS-ΔTM-BD-R	AGATGACT<br>GCAGGTCGACGGATCCTCATGTACATAATTT<br>CTTGTTCGAA	Y2H

The full length cDNA of genes in this study:

>CgRIG-I-1 (Cg24392):

AGGTGGAAAATAAACCGAAACTATCAATTTGATCCGAATGTCATCCCAGGACGACTGACAA
ACTATTTGTTATTACTGAGCTTTTGCGGTCATCGTAAAGAATATCCGTGATGTTAGCTAACGG
CCCAGTCAGTGCCCTCTCTGGGATGGTCAGTATAAGGTGTACACTAGGTACCCATGATGTCC
CGAAGCTAGTTGCTTTGTATAGGCCTCTGACCGTTCGCTGTGCCCGAGGAATAGAGCTACTC
TCTCACCTGAGGAATGCTATTGATGATTTAGGCCGTCAAGAGGTCCGTCAGATCAGCAGAC
AACAAGGGGACATTGCCTCTATGGAATGCCTGCTTAATCATCTAGAAGTCAGCGAACATCCT
AAAAAATGGGAAATGTTTGTGGATGCTTTAGAGAATAATGAGTACCACTATCTTGCAAACGC
TCTGAAAGGCCAGGAAGTGCCAAAAGATGTATTCATCAAATTTCGGAAACTCTTGATGGAA
CACAACCTTGAGTTCATCGGACGAATAAATCCAGCGGAAATCCTTCCAGAACTGCATAGCA
GGAAAGTGTTAAACGATATGGATAAAGAAAACATTACAGCAGAACAAAGGACGCAGGGAA
ACATGGGCGCCACAGGCGTTCTTCTTGATCGAGTATGGCGGCGCCATCCAAACTGGTACGA
AGAATTTTTGGACGTTCTTTGCAAATACTATCCGGACATTGTCAAAAGAATGGACACGGATT
TTTATGAGAAATGGCAGATAGCAAACAACAAGCAAGTTGAACTTCCAGTAAAACAACTTGC
CAGCTGTTCAGAAATTAACGACAGCAAAAAAGAGGATTCAGATATGGCACCACCAGAATCT
TCACCTAGCTCAAACAGATTGGATTCTTTTGGCAGGAAGAGTTCTTCCTCAATTCGTGAGGA
CTACATGTCAGCAACGGGTGATCCCCAAAATACCCTTGGTGATCGCTCCACTCCCCCACAA
GATAGCATTGCTTTTGAACCAAGTGGCGAGTTGACGTTGAATAGCTTCAACAGTGATTCTGG
TGATTATGACGATGAAATAACTCACGTAAAGAGAAGGATTTCAGAAGAACAACTAAATTCC
CTGCATTCAGTTCAGAAACGGGAACCGGAGGATAAATCTATTGAAGATTTGGGAATTCCAG
ACACGTCAGGAGGTCATACCTTGAGTTTTGTGTCAACCACAGGTTCAAGGTCAACAAATCT
GAGGGCAAACATCCAGGCTGGTGACCAGGAAAACCTGAGTAATGTTCCCTCCCTCAGCGG
CGGTGCAACTTCATCTTTGGTGGGTCGGCTGTTACAAAGTATAGAGAACTGTGTAAATGAA
GATTTGCAGAGAGAAGGGGGCGCTGTTCCCAATATCGTAGGACCTGAACATCCAATCATCG
ATACAGAGTCTGTTGACGAAAACAACGAGAGTTCAGACGAAGAAGAACGCATCCCTGCTA
AACCTCTAAACTTGCGCAGCTATCAAATGGAGTTGGCACAAGCAGCCCTGCAAGATAAAAA
CTGCATCATCGTTGCTCCTACTGGAAGTGGAAAAACACATGTTGCTATGAAAATCATACAGA
ATCATCGTGAAAAACGGAGGCGTCTGAATATACCGAAAGTCGCTTTTTTAATTGAACAAAG
CGCTCTTGCAGAACAACAGGGAAAAGTTTGCAAAGAGTATCTGGGTTGTAAAATTAAGGTT
ATCACTGGAGAGAAGCAACGCACAGAGAGCCTCCAGAGCCTTTCGTGTTGGATTCAGAAA
AAGGATGTACTGGTCATCACTGCACAGATTTTGGTGAATGCGTTGGCTACGGGGGACGTTC
AGATTGAAGCTTTCTCCTTGATAGTTTTTGACGAGTGTCACCACTCGCATGCCAAGCATCCA
TACAATCAAATAATGGCGTACTATCTGGATCTTAAGCTGGAAGATAAGCACAAGCAACTCCC
CCAAATTGTAGGTCTGACAGCTTCAGTTGGAGTCGGTAAGGCGAAAAATGAAGAGAAAGC
AATGGACTGGATCTTTAGCATGATGGCCAATATGGACGCCGAGGAACTTTGTGTTGTTGAAG
AAAATAAGGCGGAATTGGCCCAACATGTTAATATTCCAGATCAAGGTGTGGTAAAAACTAA
CTCAAGAAAGAAAAACGACTTCGGGCGAATCATAGATGGCATCATGAAGGCGATTCATAGA
TGGATGATTCAATCTATACATGCCAAGGCTCTGACGGACCAGAGCGTTCTGAAGCCCCCGG

CGGAGTGTGGCAACGATCAGTACACACAGTGGCTCAGTCGACTGTGGAAGGAGGGGGCAA
AAATAGTAGACGAGAAGGCCAGGCGCTTCATCCATTCCTGCCGAGTCAATCTGGATATGTAT
AACAAAGCCTTGATTATATATACCGATGCACGCACCTCTGATTCTCTTGCCTTCATCAAAGAT
GAACTGAAGCGTTGGGACGAACACCAAATTCCTGACGATACGGACAGAAAACTCGGATCT
TTCTTCGAAAAAACCCAAGGTAGACTACAGACATATACAATGGATCCAGATCACAACAATC
CAAAGCTGATGGAACTACGGAGGTTGATATTGCAAGCATTTGGGAATGATGCTGATTCACG
AGGCATTATATTTGTTAGAACCAGAGATCTGGTTAAAGCAATTTACCGTTGGATGATGGAAA
CGGATGATCTACGACATCTCAAACCTGTCATGTTTACTGGAGCGCAAGCCAAGAGCAGCGC
AGGAGGAATGACAAAGGTACAGCAGATTGATGCTCTAAGCCTGTTCAAGGAGGGTCGACA
TAAAATTGTCATTGCTACATCTGTGGCCGAGGAAGGTCTGGATATCCAGAAATGTAATCTTG
TGATTCGGTATTCCTACGTCAGTAATGAGATCGCCATGGTCCAGGCAAGAGGAAGAGGAAG
ACGTGAGAATGGAAAATACTTTGTGGTGGCTGAACAGGGTGATAAGACTGCTGAGAGGGA
AGAGTTGAACATCATCAGGGAAGCTATGATGAACCGTGCCATAGAGCTGCTGAGGGAGAA
ATTCAGGAGGGAGCGGAGAGAATGTCTGTACATCATACTTGGCTTGCAGAAAAATGCTAAA
ACAGAGCGTGACCTTGCGGCGAAAAATAAAGAAGGATTCTTAGTCCGCCAGGGCGAATAC
GTGCTCAGATGTCAAAAGTGTAGTAAGTACATATGTATGTCAAATGGGGTCAGAAAGATCCA
AAACGCCCACCATGCCTGTATTTGTGACGACATCAAAGAACGAGTCCTGGGGCAGCGCCTG
CCTCGCCCCCAGTTCGAGGACGTGGACCTTAAGTGTGCCGTCGGCAAGCTCCTCTGTCGAT
CCTGTGGGTCAGATCTAGGAAATATCTCCATCTACAAAAATGCCCAGTTCCCGATCCTGAAG
ATAGAGGGGCTCTTGATGGAGGACAATATGGGAAGACGAGACGTAAAGAAGAAGTGGAAG
AGCGTGCCGTTCTTGGTGAAGGAGATAACTGCTGAAGATATATCGCAGCGGGCCCGTGGAG
AGAAACTCATTGATATGATGTGAATGAATGTCAAAAATGATGGAGATGGGGCGGAATTAGTT
TAAGCAGCTTTCAGTGCATTTTAGTTTCTGCTTGCATACTGTTCTACACTTGTAATGTATTCTG
ACTTAAGACATTCAAGAATACTGCGGAGATTTACCAGTTTTACAATATAAATGTACAGTCATT
CGTTTTGCAGGACTGAATATCGTACATTTTTAACTATTTGTAAAGAGGTAGAAAAAATGAAA
ACGTATACTGTATACACAGTTATCACACATTATAAATTCTTATTTGCTGGTGCACTTTATCCTC
TTTTCGACTAGTATACGATTTTTACCATATTCAATAATTATGTAAAACATATTGCACATGTATTT
CTGTGTTGATTTTCATGTTGCCTCTATTTTCTCGTTATCTATATTTATGAATAAAATCTATATGT
AATTAGCCGTCTCTACCTGTAAATAGATTTAATGTATTTATGACATTTTTAGGCAAATGACATA
TAACAATATGTGGTTCTTTAAATGAAATGTATATTTGAATGTTGTGAACATGTTTATTTAATAC
TGAAAGCAATTTGCACAAATAAGAAACACTTCTACATGTATAATAAAGATTAGAATAGATAA
TTACATGTACACTGTTATGGAACGATCTGCCTCTTAGTAAAAAACGCGTTTGTGAAAGCAAG
AAGCATTGGCCACGTCGTGTAGACATGGCATTATGATTTATCTTAAATGTACAGTATTTATAAT
ATTTTATGAATAATTGTGATAAAACTGTCTTTCAATTTGTTATACATTATGTATGCACATTCATT
TGATGAAATAAGTTAGTGAATTGATGAACACAACATGTACATGTATATTTGAACTATTGTGCC
ATTGTCTTACAGATATTTTCAATAAAAATGAATTAAATAAAAAAAAAAAAAAAAAAAAAAA
AAAAAA

***>CgMAVS*:**

TGGATTTCTTTACTTTCGTTTTTGAGTTTGCCTGTGAAACTTCTGATAATGATGTAATGGAGA

GATTGATACTACCCCAAGAAACTACTTGTACATTTCCGTACGACTAATAATGCCATTCCAATC
GATCAAAAAATAGACCAGAATGTCAATTGAAGATGACTATCGCAGACGTGTAATACAGGAA
AACTTGGTGAAACTTGTTGACCGTTTGTCACCTGATGAATTATTGCCCCACCTTTCATGCCT
ATCAAGGCAATCACAACAAAAGATAAGATGTCGGTTGGCACAAGAGGGTCAACAAGCAGC
GACCGTTTTATTATTAGACGACTTGCCCAGAAAAGATAATTGGTGGGAGCAGTTAATGTCAG
CTTTATGTCATCCTGCTGTTGGTCAAGTGGATATGGCAGAGTTACTACAAACGCAAGAAGAC
ATTTTTCGTGGAATTAACACGAAATCTGAAAAATTAGAGTTAATCGCCGAAGTTCATAAAGG
AGCACCACAGAATGAGAAGATGCCTCCATATAAAACGAGAAAAGTGGAAATAAACTGGTC
TACTCCTCTCCAAAAATTACCATTTTCTGTCGTCAAAGTTTTACAGAGATTAGATGTTGACTC
AAAATGGAAAGATTTGGCTGCACAAACAGGTTATACAGTGGAACAGGTGGACAGTTTGGA
AGCATATACAGACAAAGACGGAGGCCACGTTCAAAAACTATACAGAGATCTTCAGACAAG
ATACAATTTCACGTTATGTCAACTTGTAGATGCTCTGCAGAATATTGAAAGATTAGATATTTT
AGATGAGCTTAGTCATATTGAAGAATTTAAACATTTAGAATGGTCCAAACGAATGACTCCTG
AGAGTTCATTTGAAAACCAAATCTTGCAGCCTTCAATTGATAATCAGATTAAAACGGCGCTA
CCGCAAAACATACAGTTAATAAAGAGAGATGAAAACAAATCGGACTGCACATACCAGAAG
GCAGAGTGTAAAAGTGATGTTTCAAACAGAGAAGTTTCGAACAAGAAATTATGTACATCTA
ACGATGAAAGTAATTCTGAGAGTTCAGTGAAAAGAATTGTGACAGCTTTAGGAGTGATAGC
TGTATCTTCTTTTATTTTTCATCGCTGCGTTTTCAGATAGAAAAAAATGTTTGTTAATATAATC
CTCAGAAATAAAGACAAATATTCAAGGTTATTTTCCATGGGTTTTTTCAAATTGAAACATGA
ATTCCAGTGCTTTAGACTACAAATTTATTATGATTTTCTTAATATGTAATACAGTATAACGTAC
TCGTTGTTTCTGTTTTTGTTTAAATAGTTGTAACTTTAAACGTTTTTGTCTTCTCCATACCTCC
AGGTTTTTTTCACATTTTGAACACATTCAGAACCACTATGCCATTTTAAAAAAACTTTTGGA
ACAAAGAACAAAGAACAAAGAAACCAAATGTGTTTGAATAAATTGCCAGACCAGAATATTT
TTTTTTTAAATGTTTTAATGAATCTCTATGAAGAAAAGCAATATGTAACACCGATTTTTGTTG
ATAATAAGCAGAGTAGACTTAACAAACCCATTTCAGAATAGTTCTAAAATGTTCATGAAAAA
TAGGGGTATGTTTATTAGGCAATTTCCGGTTGCAGAAAAAGATATTAAAAAACAGTTACAAA
AACAATTACTATAACTGTCAAAACGTTACACAGAAGGAAAGTCCATTTTCCTTCTATGTAAA
AATTACCATGATAATGATAACTAAAGTGACATTTACTTGGAAGTTTGAGCCCATCCCCTGCA
AGTCAGTGCAACCCGTGCACTCGATGTACATGTACAAGTAGCATTAATAGAATCACTCAAGA
AAAAAAAATCAAATTCAACATCATAATTACAAATTAAAAGACTAATGAAAGATATGAAAATC
TTTAATTCTCTTAAGATAATTTTAAAAAGAACTACATGTATGTGTAGGATAAAGAAAAAAAAT
CAAATTCAACATCATAATTACCAATTAAAAGAATAATGAAAGATATAAAAATCTTTACATCTC
TTAAGATAATTTTAAAAAGAACTACATGTGTGTGTAGGATAAAGTGTTTTATCCCCCCAAATT
CAGAATTATAAATAGAGCTTTAGAAATTTGCTAAAGAATAGGTTATATATATCTACATGTAGTT
CAGGTTTATAAAGATAAATTGTCTAATCGAGGTCATAAAAAAATATGAAATGTTAATAATATG
CTCAGTTCACAAAAAGTACGTATTTTATAAAAAAAAAAAAAAAAA

>*CgTRAF6* (*Cg10206*):

TGTACAATTCTGACAACTAGTTTTTGTTTAAAATTGGACATTGAGTATTCATTGGACGTGGTT
ACTTACATATGTAATTTTGAAATGTAGTTTCTACCATGTAGTATAGGTGGATTGTAGGTGGAA

TCATTAATAACCAATTGATTGGCAAGCTGTTTGATACATGTTTTGGAGGAACCACTTTCATGA
TATTAATAAAGAAATGCTCTGTGGACTAAGGAATCAACATTCACTACAGTACAGAACAAAA
GACATTGCCACAGAAATCATCCAGGATTGATTTGCCTTCAAATTTACCAACAGAAAGACATT
CTTGTTGTGATGATTTAATGGATGAACCTTTGATTAGCTGACCTTTACACTGTTGCCTCAAAA
ATTGAATGCTCAGAGAAACGATAGCAAGGATGCATGTACAGAGAAGGGGTTCCAAGAGGA
CCGTGTATTGCACGTAGTACAAAAAATGACGTCAGAGCGACCTTTGTCGTCTGGTCTCTATG
AGTATACCAACGAGCCCCGCCATTCATCTCCCGTCAGTTCCGGAAGCTTTACTTCCGGAGAG
GGGCTTTCCGGTCGGGAGGAGGGATATGACTTTGATTTCATCATCAGAGACGAAAAGTACG
ACTGTCCAATTTGTCTCCTTGTTTTGAGGGATCCATTGCAGACGACATGTGGACATCGCTTT
TGTAAAAACTGCATCAACAAATGGCTCAAGGAGTCCGACCAGAGGTGTCCGATAGACAAC
ATGCCGATCACAGAATCCCAACTGTTTCCGGACAACTTTGCGAAGCGCGAAATTCTGGGCC
TCAGTGTCAAATGCCCCAACTCCAAAGAAGGCTGTCAAGTGATCGAGACTTTGAAAAATAT
CCAGAGACACCTTGACGAGTGCCAGTACGTGCCCATACCCTGTCCCAACAGATGCAGCCAC
ATCCTCCTCAGGAGGGACATTCAGGAGCACCTATCCCACATCTGCCACAAAAGAACCCTCA
TTTGTAACCAGTGCAGCTCGGAGGTGTTAGCTGAGGAGATGCAGGAACACGAAGATGACC
AATGCCCCATGGCAATGGTACAGTGTCCGCACTGCGCCATGGAGTTAATGCGTGAACAGCT
GCAAAGACACTATGACCACGATTGTATGCGGCGATCCATAGACTGCGTTTACTCCAAGCTAG
GCTGTAGTGTAGGAAAGATTCCTAGAAGCGAAATGGGCAAACATATTCAGGAAAACCTGCA
CAACCACATGCAGCTTATGTGTCAAGCATTAACCAACATCCACCGGCGGCTAAACATTCCCG
CCAATAATCAGCTCACCCACAGCCATTCCTTACCCGACCGAACGGAACCATTCACGCCGGA
AGACAGGCGCATGGTCGAGGGCATCGGCAACGGACTAAGCCACGCAGTTAATCTCTTGCAT
TTATCCGATAATCCATCACTCGCAATACCTCAAGCAGGACCACATTCTCTCGACAGCTTTTGT
CCACCGGATACAAGCCTTCGGCAACTGCGCGGTGCCCAGGGAAGTGACGAAAGATTCGGA
AGTGAACGAACGCAAATCGATACAGAACTTACTTATACCTCAAATCCGTCTAACAATTTAGA
GGGGTTTCCTAGAGTCCCTTTTGATGATGAATCCCAGTCGCTAAAAAGTCAGAACTTATCTC
AAGATGAGAGTTTAGCAAGGCACGAGTCGGCACTTCATGACATGAAACACAAGGTGGAGT
ACCATGAAAAGAATAACGCTGCTCTAATTAAAAGAGTAAAAAGTCTGGAGAACGCCCTCAC
TGAGCTGGAAGGGCGGTGTAGCAATGGTGTATACTTCTGGAGAATCAAAAGTTATTCAAAG
TTTAGAAACGAGGCCGAGTCGGGCGAGGTAACGGCAATTCATAGTCCGGCGTTTTATTCTA
GTTGTTTTGGATATAAAATATGTATTCGCGCAAACTTAAACGGAGTTGATTCCGCCAGAGGA
ACACATCTCTCTATATTTGTTCATTTTATGCAAGGTGAATATGACGATATCCTGGAGTGGCCC
TTTAGTGGTCGTATAATGTTAAGCGTTCTGGATCAGAACCCGACCTGTGAGTTACGATCGCA
CGTGATGGAGACTTTGGTCGCCAAGCCAACGCTGGCAGCCTTTCAGCGCCCCACCACCCCT
CGTAATCATAAAGGCTTCGGCTACATGGAATTCCTTCCACTAAGTGTCTTGGATAATTCTTCG
TACATTAGGAATGATACTTTGATAATCAAAGCTCAGATCATTGGGAGATCGGAGTGAATGTA
GGAGTGCGCTTGTAATACCCTGGTAGTATTATTATTATCATAGTGTGATAACTCAGTTTGTGA
AACCATTACTTTGTCAATATTTAAGTTGATTGTGTATATCATTAACAGAGAATGCTTGATTAAA
CCCTGGGATATGTATCAAAAAAAAAAAAAAAAAAA

>*CgIRF2* (*Cg21171*):

ATATAACTGATACTAACGTAGACAGTGAATGACAATTGGTATTTAACTTTGATTCCCGTGTGA
TTTAACAGGTGCAATCAAGATGGTGCAGAGAAAGCAAAGTCAGCCGGTTATTAACCAGAG
AAAACCAATAAGGCCCATAGAGCGACAGAAAATGAGGCCATGGCTGGTCAACCTTCTTAAC
AACGAGAAGGTTCATGGATTTTCCTGGGTGTCCAAGGATCACGAGACTTTCCGGATATCTTG
GAGACATGCAGCCCGCCAGGGATGGGACCCTATTATGGACGCCGGACTCTTCGAACGCTGG
GCCAAACATACGGGAAAGTATGTAGATGGAGATGAGCCCGACCCTAAGAGGTGGAAGGCC
AACTTCCGCTGTGCCCTGAATAGTCTACCAGACGTCAAACAGCTCAAGGACCAAGGCCAG
CGAAAGGGCAAGGACGCCTACAAAGTGTACCAGTTTTTGAACGAGCGGAAAAGTCTCCAG
AAACACAAGACTGCAGAACGAACGACAGTGCCAAAGGTCATACAGACCAAAACTAAGAG
GAGTTTGAGAGCCCGCAACACAAAGAAGATCTCGTACGCCAAGATGATGGCGATGGACAG
CGAAGGGGAGGAGGAAGAGGAGTCTTCTTCAGACTTCAACGCCTCAGAGTCGGACTGTTC
CACATCTGCAGGGAACCAGACCCCTGAGCGGATGGAGGAGGAGGAGGAGGCTTTGGACA
GTGATCCTTGTTATGAGGAGGGACTGGAAACACGTCTCCCAGACTTCAACAAAATCTGTAA
ATCCACCGACCTCACAGCCCTTCAAAAGTACCTGCCTTCTGAGAAGGACTTAAGGACCAAT
TTAGCCTGCACCGTCCAAAGTGCAAAACAGACTTTTCCTTTCCTGCAAGATACTCCTATGGC
CATAGGAGGGCAAGAAGAAGATGATTCCACCTCCACAAGTAGTGAATTCACTAGCGAGGA
ACTCATCCAACTGGTCATGGATGCAGACGCCCAGGAGCAGTTCTCAGACACCAACTTTACA
GACTTATGGGGGAGTGGACTTCCGGTGCCCCAAGAGGTCGCTTATGTCGACGCAATCATTG
AGGAAGGTGAGACGACCTACTTTATTTTGGACAATGTTGAAAACGAGGTTGTGATCACTGA
TACCCCGGCGGTGCGCTGTGAACAGATAACCAATGGCACAGACGTAATGCAGTACACCGGT
CTTCGCATGTCCCAGTTATGAACGAGGTCTAGTGGCACTCATTGACTATTGTGATAACCCCC
AATTATCATCAGTCGGATGGTTCAACAAGAAGAGGCCGGCCAAGTGTATTCATCCGCCATTC
TCCGTTCTGAATAAATATATAAACGTTTTCATCTTTCATCGTTCACCATGTAGTGTATCCGGTG
ATTTTATGAATGGTGATTTCTTTTTATCGCCTTTGTGGACAAAGGAAGCAGGTCTCAAATGTT
GAAGTGGAATTATTTGTGCAATGTAAGCAAATTCCGGCCGATTTCGTTCCGCCCAGCTGCAA
TTCGAAAAGTACTCCACGACCTAAACAGCGCTATACCGAAAAAATGCTCAGAAAAAAATAA
AATGCAAGACTATAGTGACTATTAACAGTTTAAAACTGGGCGTGCTTCTTGCAAAGATTTAT
ACATGTTATGATGTATTTTACCGATTATATACTGTATGTACAGTTTTGATGAAATACATGTACA
CTGTACAATTATTATATGTTTGTTGAATAAAATATTGAAAATTGAAAAAAAAAAAAAAA

>*CgIRF8* (*Cg03270*):

AGAGTTAGCCGTATCTTCTCGTAACTTTTACCTCTTGTCGACATGGCAACAGAAATTGATATT
CGCAGATTTTTAGAGGTCACCATGGACAGTGGTCAGACACGACAGAGAATGAGGCCGTGG
CTCGAGTCCAAAGTGGAGTCTGGACAAATTCCGGGTCTGGCCTGGATAGACCGGGAAAAA
AAGATCTTCAGGGTTCCCTGGAAACACGGGGGCAAGCATGACTGGAATGAGGCAGACTCC
ACAATATTCAAGGAATGGGCCATACACACGGGCCGTTATAGAGAGGGGGTGGACCATGCAG
ACTGGCCCACCTGGAAAACACGATTCCGCTGTGCCCTCAATAAACTCCCTGATATCCAGGA
AATGAAGGATCACAATCAGCTGGATGGAAGTGATCCCTACAGGGCCTATAAGTTCCTCAGTA
AAGAAGAACTTACTGAGATGAAATCCAGTAACAAAGAATACCGGGAGGTTTGCACTTTAAA
TAAGGCTTCAAGTCCCAAGGAAATAACCTCGTCTGATGTTATAGTACTCTCGGAGGTGAAA

CCTGTGTCTGAAATGCCAAACTTTCCATCTGATCTAGAGGATTTGATGTTGGAACCAATAAA
GTCTGATCCAAATCTTGCACCAATGATTCAGTCAGAATTAATCCCAACTCCAGCAGATAACA
TAAAGGAAGAACAAGGGGATGAGGATATGAAAGATCTGTGTGAGGAGTATACCCAGATGAC
AACCCGGGGCCCAGCACTACCCTTGATATCCCCGCAGATGCCATTCTTGCAGGACCCTGCA
GACCATCAAATGGAGATAATACTTCGATATTGCCAACAACAAGTTTGTAATTACGGGGTCTG
CAGTCCGTATGGATGTCGGCTTTTCTATGATCCGGAGAGGAATGACCAGTTTGTAGATGGTG
CCATCAAACAGGAACTCTTTGGACCAGAGCAGTTTGAACAGTTACAGTTCCCCACCTGTGA
AACAAGCAACCCTGGCCAGAGGGAGCACACTTCCCAACTTTTGATGGCGTTAGACAGGGG
GCTGCTTTTGGAGTGCCATGAAGGCATTATCTATGCAACTCGAAAATCCAGATGTGTTATTTT
TATCTCGTCTCCTTGTGTTAACAATGGTGAACCCTTTAAGTTGGAGCGGAATTCAAGAACGG
CAGTCTATGATTTTCTCAAATACTTTGAAACGGCGCTACAGCAATATTTGACTGGAGCTGCC
AAGAAGCCTGTGGCCCACTTTCTCGTGGGATTTGGTCAAAAACTGCCACCAAACAATACTG
ACCTTAGTGGCCTGCTAATTTCAGCTCAGGTGTTCCATAGCAGAGCCAAACATCGCCTGAAT
CAGGTTGCTTGTACAAGTCCTTTGTCATCCTCCATCAAAATCTCCATGCCGGATGATTTTGAT
AGGATTTTAAATTTTGTCAAAGACAGATGCCATGGACAAATGGAAACCTGATCATAACGCCC
AGAGTGTGGATCCCTTGTTTC
